# A survey in natural olive resources exposed to high inoculum pressure indicates the presence of traits of resistance to *Xylella fastidiosa* in Leccino offspring

**DOI:** 10.3389/fpls.2024.1457831

**Published:** 2024-09-30

**Authors:** Pierfederico La Notte, Maria Saponari, Soraya Mousavi, Roberto Mariotti, Raied Abou Kubaa, Roya Nikbakht, Giovanni Melcarne, Francesco Specchia, Giuseppe Altamura, Angela Ligorio, Donato Boscia, Antony Surano, Pasquale Saldarelli, Annalisa Giampetruzzi

**Affiliations:** ^1^ Institute for Sustainable Plant Protection, National Research Council, Bari, Italy; ^2^ Institute of Biosciences and Bioresources, National Research Council, Perugia, Italy; ^3^ Forestaforte, Frantoio Oleario Giovanni Melcarne, Lecce, Italy; ^4^ Centro di Ricerca, Sperimentazione e Formazione in Agricoltura “Basile Caramia”, Locorotondo, Italy

**Keywords:** Xylella, olive, resistance, SSR, genotyping, parentage analysis, transcriptomic

## Abstract

**Introduction:**

The epidemic spread of the harmful bacterium *Xylella fastidiosa* causing the “olive quick decline syndrome”, decimating olive trees in southern Italy, in the region of Apulia, prompted investigations to search for olive genotypes harbouring traits of resistance.

**Methods:**

A prospecting survey was carried out to identify, in the heavily infected area of Apulia, olive genotypes bearing resistance. Given the limited genetic diversity in the commercial olive groves with few cultivars widely cultivated, surveys targeted predominantly spontaneous olive genotypes in natural and uncultivated areas. Trees, selected for the absence of symptoms, were subjected to diagnostic tests and parentage analysis to disclose their genetic background. Transcriptomic analyses were also employed to decipher the molecular pathways in resistant genotypes. Artificial inoculations were carried out to confirm the resistant phenotypes of four open-pollinated seedlings of the cultivar Leccino.

**Results:**

Among the 171 olive collected genotypes, 139 had unique simple sequence repeat (SSR) profiles, with the cultivars Leccino, Cellina di Nardò, and Ogliarola salentina being the most frequent candidate parents. Among the Leccino progeny (n. 61), 67% showed a highly resistant (HR), resistant (R), or tolerant (T) phenotype to infection by *X. fastidiosa*. The occurrence of such phenotypes among those deriving from Cellina di Nardò and Ogliarola salentina was 32% and 49%, respectively. Analyses of the transcriptomic profiles of three Leccino-bearing genotypes, naturally infected and not showing symptoms, unravelled that a total of 17,227, 13,031, and 4,513 genes were found altered in the expression, including genes involved in photosynthesis, cell wall, or primary and secondary metabolism.

**Discussion:**

Indeed, transcriptomic analyses showed that one of these genotypes (S105) was more resilient to changes induced by the natural bacterial infection than the remaining two (S215 and S234). This study consolidates the evidence on the presence and heritage of resistance traits associated with the cv. Leccino. Moreover, valuable insights were gathered when analysing their transcriptomic profiles, i.e., genes involved in mechanisms of response to the bacterium, which can be used in functional genetic approaches to introduce resistance in susceptible cultivars and initiate strategies in olive-breeding programs through marker-assisted selection.

## Introduction

The cultivated olive (*Olea europaea*, subsp. *europaea*, var. *europaea*) is one of the most important oil crops in the world, and 95% of total olive oil production is derived from the Mediterranean Basin (Mariotti et al., 2023). Olive accounts for a very rich varietal heritage, which is represented by more than 1,200 named cultivars, over 3,000 minor cultivars, and an uncertain number of genotypes including pollinators, local ecotypes, and centennial trees (Hosseini-Mazinani et al., 2014; Diez et al., 2015; Mousavi et al., 2017; El Bakkali et al., 2019; Salazar-García et al., 2019; Sion et al., 2019; Valeri et al., 2022; Mariotti et al., 2023). Nowadays, the olive cultivation scenario is rapidly changing with the transition to modern and intensive cultivation schemes, entailing the plantation of a restricted number of cultivars suitable to such management systems, thus reducing the large biodiversity typical of the traditional olive industry. Locally grown varieties, empirically selected by farmers and naturally tested for their resilience to climate change, reduced water supply, energy, and chemical resources, represent a high-value source of variability for the sustainability of olive cultivation (Sakar et al., 2016; Sorkheh and Khaleghi, 2016; Cheng et al., 2017; Veloso et al., 2018; Hussain et al., 2019; Khadari et al., 2019; Mousavi et al., 2019; Atrouz et al., 2021; Islam et al., 2021; Omri et al., 2021; Topi et al., 2022; Valeri et al., 2022; Duran et al., 2022). In traditional olive-growing areas, such as Apulia, much of the great genetic and phenotypic variability represented by the local germplasm is only partially explored, and often, some traditional varieties have been grafted with a few selected cultivars as happened in the Salento peninsula with the cultivars Ogliarola salentina and Cellina di Nardò. For this reason, it is important to conduct an in-depth investigation to adequately preserve and evaluate the current diversity of the olive tree and thereby initiate a renewal of olive growing against emerging diseases and climate change.

Currently, this important Mediterranean crop and landscape iconic tree is threatened by a severe disease, the olive quick decline syndrome (OQDS), caused by a strain of the bacterium *Xylella fastidiosa* subsp. *pauca* (*Xfp*). This harmful plant pathogenic bacterium has been extensively investigated in the American continent where the pathogen originated and has evolved under different selective factors in biologically and genetically distinct populations (Castillo et al., 2020; Kahn and Almeida, 2022). In the Americas, it is well known as the causal agent of detrimental diseases in crops and landscape trees, while it is only in the last decade that outbreaks emerged in the European and Mediterranean countries, with infections affecting olives, almond trees, and several essences typical of the Mediterranean flora (Landa et al., 2020). Bacterial invasion in these new territories encountered favourable climatic conditions, a number of susceptible host species, and efficient insect vectors, such as the spittlebug *Philaenus spumarius* L., contributing to the expansion of the initial outbreaks and favouring the persistence of the infections in endemic or epidemic forms. OQDS is the most emblematic example of the detrimental impact of this pathogen conquering the Old Continent (Saponari et al., 2019). This deadly disease was described for the first time in the South of the Apulia region (southern Italy), where in 2013 an outbreak of *Xfp* was recorded (Cariddi et al., 2014). In the same area, the extensive cultivation of highly susceptible olive cultivars Cellina di Nardò and Ogliarola salentina caused the rapid evolution of the initial outbreak into one of the most severe epidemics in the history of plant diseases. Several million olive trees, including centennial plants, have been decimated with inestimable damage affecting different ecosystem services (Schneider et al., 2020).

Further investigations in other olive-growing areas in the world disclosed the occurrence of symptoms resembling those of OQDS reported in Apulia, Brazil (Coletta-Filho et al., 2016), Argentina (Tolocka et al., 2017), and Ibiza (Moralejo et al., 2020). In all cases, different *X. fastidiosa* (Xf) strains belonging to subsp. *pauca* (*Xfp*) have been detected and/or isolated from the diseased trees. Conversely, in California where only strains of the subsp. *multiplex* have been reported infecting olives, only mild shoot dieback phenomena have been so far described (Krugner et al., 2014).

Nowadays, no curative solutions exist to rehabilitate *Xylella*-infected plants, and currently, the control relies on reducing vector population and removal of infected sources, whose success depends on the timely application of the interventions (Morelli et al., 2021). In this context, as for many vascular plant diseases that are difficult to control with conventional means, genetic resistance represents the most promising long-term strategy for their sustainable management. With reference to the major *Xylella*-susceptible crops, such as grapevines, citrus, and olives, significant variations in the plant response and transmissibility of the bacterium are observed among genotypes and cultivars. Plants of susceptible cultivars generally harbour high bacterial populations in all tissues and show severe symptoms, while those exhibiting resistance maintain low bacterial loads, erratic distribution of the bacterium in the infected tissues, and limited expression of symptoms. In grapes, all domesticated grapevines (*Vitis vinifera* ssp. *vinifera*) are susceptible to Xf, whose infections cause the notorious Pierce’s disease (PD), while some wild relatives, mainly *Vitis arizonica*, exhibit strong resistance to PD. Resistance phenomenon in this wild species has been associated with the segregating *Pierce’s disease resistance 1* (*PdR1*) locus (Krivanek et al., 2006; Riaz et al., 2008).

Similarly, in citrus, the existence of genetic traits of resistance to *Xfp* has been reported; i.e., several mandarins (*Citrus reticulata*) are considered resistant (Niza et al., 2015), while the majority of the commercially cultivated sweet orange (*Citrus sinensis* L. Osb.) varieties are susceptible (Laranjeira et al., 1998). Citrus hybrids obtained between these two groups of species demonstrated the segregation of the resistance genetic traits in the progeny (Mauricio et al., 2019).

In olives, resistance phenomenon to *Xfp* has been discovered in two cultivars, Leccino and FS-17, whose infected trees show limited occurrences of desiccation phenomenon on the canopies, together with low populations and localized presence of the bacterium in the canopies of the trees. Recent studies, mainly based on physiological and anatomical observations and metagenomic approaches, suggest a quite complex network of host–pathogen interactions and factors involved. For example, resistant trees of the cv. Leccino appear to be able to isolate the bacterium in xylem vessels (Walker et al., 2023), respond to and better manage the drought stress caused by the bacterium (de Pascali et al., 2019; Surano et al., 2022), efficiently resist to the activity of the bacterial cell wall-degrading enzymes (Montilon et al., 2022), and sustain a “resilient” microbiome (Baptista et al., 2019; Giampetruzzi et al., 2020; Vergine et al., 2020).

It should be remarked that despite the relevant research investments over the last century, the genomic architecture of resistance to *Xylella* has been limitedly investigated, and its genetic basis remains largely unclear. Even so, the most recent research outcomes suggest the existence of common defence responses across the main crop species. For example, investigations on the resistant olive cv. Leccino indicates that proteins belonging to the leucine-rich receptor kinase family are involved in the plant response to infections (Giampetruzzi et al., 2016), a research route that is supported by the existence of similar evidence in grapevine (Agüero et al., 2022) and citrus (Rodrigues et al., 2013).

In olives, despite the germplasm richness in terms of genetic diversity, preliminary results of large-scale screening for *Xfp* resistance indicated that none of the tested olive cultivars/selections showed a level of resistance similar to or higher than that of Leccino and FS-17 (Boscia et al., 2017). In contrast, in olives, the availability of large, crossbred populations of genotypes is very limited due to scarce investments in breeding programs, whose main challenge is the long duration of the preselection and selection phases. However, the emergence of OQDS makes it no longer possible to devote research efforts to the development of new resistant genotypes by classical or biotechnological approaches. In the meantime, to take advantage of the natural selection process of resistant genotypes in the area affected by the *Xfp* epidemic, we conducted an extensive survey in the demarcated infected area of Apulia where the cultivated and natural olive germplasms are under high pressure of inoculum for many years.

Surveys carried out over the past 6 years allowed us to identify 171 *spontaneous* olive genotypes, based on the absence of manifest *Xfp* symptoms, which were tested for several years to monitor the occurrence of the infections, the bacterial population size, and the development of symptoms. Over time, several genotypes became symptomatic and were discarded from further studies and assessments, while many of them remained symptomless or with mild desiccation phenomenon. Parentage analysis was carried out to disclose the genealogy of these genotypes and assess which cultivars contributed to the resistant phenotypes. On a few selected genotypes displaying highly promising resistant phenotypes, more in-depth analyses were carried out, i.e., transcriptomic profiling and artificial bacterial inoculations to reproduce under controlled conditions artificial infections and monitor the genotype response to *Xfp*.

## Results

### Phenotype assessment on the selected germplasm

In 2022, a final assessment was performed on all selected spontaneous trees by scoring the presence/absence of symptoms and by testing multiple sub-samples from each tree. Genotypes were categorized as highly susceptible (HS), susceptible (S), tolerant (T), resistant (R), and highly resistant (HR) based on symptoms severity, estimation of the bacterial population in the tree, and frequency of *Xfp*-positive shoots in the canopy. According to the results of the genetic assessment, these 171 genotypes have been divided into two groups after genetic analysis—open-pollinated seedlings (n. 139) (**Supplementary Figure S1**) and cultivars (n. 32) (**Supplementary Table S1**)—with the latter including genotypes visually misidentified as putative seedlings.

The selected genotypes were all asymptomatic and with low or undetectable levels of the bacterium when firstly identified in the area (**Supplementary Figures S1A, B**). Nonetheless, almost half of them during the period of observation (2016–2022, **Supplementary Figure S1B**) became symptomatic and highly infected, as shown by the number of genotypes categorized as HS or S (**Supplementary Figure S1E**). However, it should be considered that concomitant biotic/abiotic stresses in some cases may have exacerbated the desiccation phenomenon.

In contrast, the absence of symptoms and the low infection rates in the remaining genotypes not necessary can be associated unambiguously with the host response, given the variable epidemiological conditions occurring in the different locations (i.e., adverse conditions for the vectors) and the observations limited to single unique individual trees without biological replicates. This shortcoming emerges also from the phenotypic assignment for the 32 genotypes that afterwards have been assigned to known cultivars (**Supplementary Table S1**). In this batch of trees, while data are consistent for the resistant cultivar Leccino and the susceptible cultivar Cellina di Nardò and Ogliarola salentina, there are examples like Arbequina and Simone whose trees have been categorized as resistant, but recent data from commercial groves and experimental plots indicate that they are among the most susceptible (Saponari, personal communication).

### Genetic diversity and differentiation analysis

**Figure 1:**
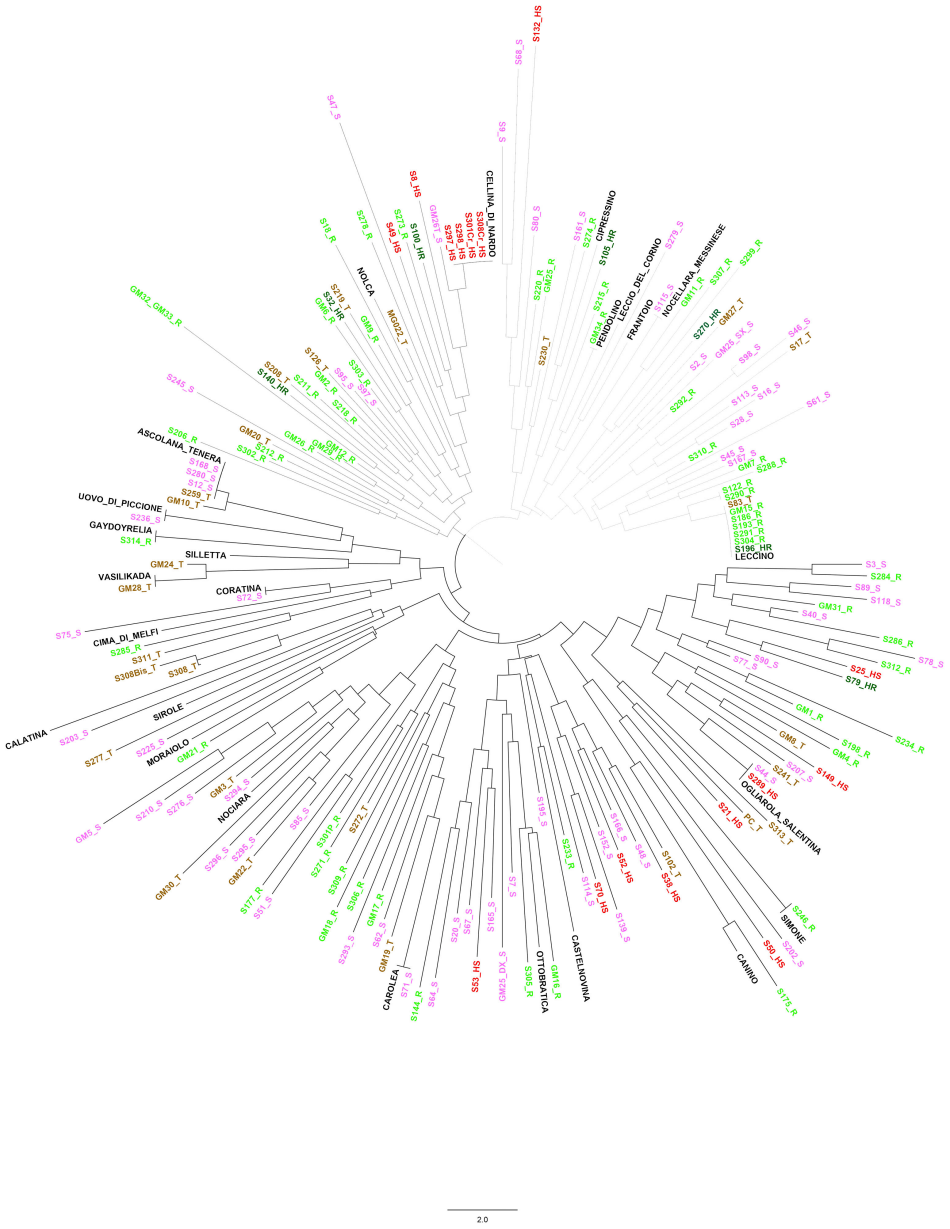
Neighbour-joining (NJ) tree based on genetic distance matrix of the 171 selected genotypes. Three distinct clusters were highlighted: the first cluster (dark grey branches) was divided into two sub-clusters; the second cluster (grey branches) with the presence of the highly susceptible cv. Cellina di Nardò; and the third (light grey branches), which contains cultivar Leccino. More details in the text.

Among the 171 identified spontaneous genotypes, 139 had unique simple sequence repeat (SSR) profiles, while 32 of them were known olive cultivars. To analyse the genetic diversity of these spontaneous olive trees, 25 mostly diffused cultivars in the Apulia region were included in the study for SSR profile comparison. A neighbour joining (NJ) statistical method, selected to draw a tree based on a genetic distance matrix, showed that spontaneous genotypes separated into three distinct clusters (**Figure 1**). The first cluster (highlighted by all branches in dark grey colour) was divided into two sub-clusters, of which one was represented by highly susceptible (nine, red colour) together with a mix composed of tolerant (12, brown colour), resistant (22, light green), and susceptible (35, fuchsia) genotypes with the presence of some known cultivars such as Ogliarola salentina, which is considered nowadays the most susceptible cultivar, along with Canino, Ottobratica, and Coratina. The other sub-cluster instead mostly includes tolerant (five) and susceptible (six) genotypes together with the cv. Ascolana tenera. The second cluster (grey branches) includes genotypes with different responses to *Xfp* and was represented by the presence of the highly susceptible cv. Cellina di Nardò. This cluster groups spontaneous olive trees both highly susceptible (six) and highly resistant (three); a considerable number of resistant (16) and tolerant (5) genotypes are also present. The third cluster (light grey) contains approximately the same number of susceptible (16) and resistant (19) genotypes, including the cultivar Leccino (**Figure 1**). These findings showed high genetic diversity and admixture in terms of susceptibility among spontaneous genotypes rescued in a limited area.

The 10 loci used in this study showed a high degree of polymorphism among the 171 unique genotypes and revealed a total of 146 alleles. Allele numbers for all samples ranged from a minimum of five to a maximum of 21, respectively, at EMO90 and DCA9 loci (**Supplementary Table S2**). The number of effective alleles ranged from 2.72 to 6.82, and Shannon’s information index ranged from 1.31 to 2.22. The mean of Ho was the same as that of He (0.80). Fixation values (F) were negative on average, excluding GAPU103A and UDO-043. A negligible or moderate number of null alleles were observed except for the latter two loci, for which the number of homozygous alleles was high. The polymorphism information content (PIC) values were higher than 0.5 at all loci, with an average value of 0.74 and maximum discrimination power for DCA9 (0.83) and UDO-043 (0.84) (**Supplementary Table S2**). To better study the allele frequency and its possible correlation with *Xfp* resistance, the 171 genotypes were divided into two populations (POP): susceptible (including HS and S) and resistant (containing HR, R, and T) genotypes (**Table 1**). The fixation index (F) had the highest value in GAPU103A of POP susceptible (0.19), unravelling the high inbreeding level in this population. A similar result was observed also for the UDO-043 locus, whose F value (0.10) was higher in susceptible POP. In addition, the frequency of private alleles had the maximum level in this POP (0.038) (**Table 1**).

**Table 1:** Indices of genetic diversity of resistant and susceptible populations (POPs).

Locus	Population	N	Na	Ne	I	Ho	He	UHE	F	PA	PAf
**DCA3**	Resistant	93	12	4.77	1.84	0.86	0.79	0.79	−0.09		
**DCA3**	Susceptible	78	11	5.72	1.95	0.79	0.83	0.83	0.04		
**DCA5**	Resistant	93	11	3.04	1.40	0.74	0.67	0.68	−0.10		
**DCA5**	Susceptible	78	9	3.45	1.50	0.77	0.71	0.71	−0.08		
**DCA9**	Resistant	93	16	5.92	2.08	0.95	0.83	0.84	−0.14		
**DCA9**	Susceptible	78	17	6.57	2.24	0.94	0.85	0.85	−0.10	168	0.019
**DCA16**	Resistant	93	16	4.33	1.91	0.82	0.77	0.77	−0.06		
**DCA16**	Susceptible	78	17	5.65	2.12	0.87	0.82	0.83	−0.06	158	0.019
**DCA18**	Resistant	93	14	4.80	1.95	0.85	0.79	0.80	−0.07		
**DCA18**	Susceptible	78	13	6.59	2.07	0.96	0.85	0.85	−0.13		
**EMO90**	Resistant	93	5	2.66	1.15	0.62	0.62	0.63	0.00		
**EMO90**	Susceptible	78	5	2.77	1.15	0.65	0.64	0.64	−0.02		
**GAPU71B**	Resistant	93	7	2.81	1.26	0.70	0.64	0.65	−0.09		
**GAPU71B**	Susceptible	78	7	3.09	1.34	0.64	0.68	0.68	0.05		
**GAPU101**	Resistant	93	12	4.62	1.81	0.88	0.78	0.79	−0.13		
**GAPU101**	Susceptible	78	10	4.52	1.79	0.87	0.78	0.78	−0.12		
**GAPU103A**	Resistant	93	11	4.57	1.74	0.81	0.78	0.79	−0.03		
**GAPU103A**	Susceptible	78	16	4.46	1.91	0.63	0.78	0.78	**0.19**	148	**0.038**
**UDO-043**	Resistant	93	16	6.70	2.07	0.82	0.85	0.86	0.04		
**UDO-043**	Susceptible	78	14	5.71	2.02	0.74	0.82	0.83	**0.10**	218	0.026
**Mean**	Resistant	93	12	4.42	1.72	0.80	0.75	0.76	−0.07		
	Susceptible	78	12	4.85	1.81	0.79	0.77	0.78	−0.01		

For each simple sequence repeat (SSR) locus: number of individuals in each POP (N), number of alleles (Na), number of effective alleles (Ne), Shannon’s information index (I), observed heterozygosity (Ho), expected heterozygosity (He), fixation index (F), presence of null alleles (Fnull), private allele (PA), and private allele frequency (PAf). In bold the highest values of Fixation index (F) and Private Allele frequency (PAF) are reported.

To identify the genetic relationships of these 171 selected genotypes with international cultivars, their SSR profiles were subjected to phylogeny reconstruction through NJ method analysis together with a set of 482 SSR data from cultivars representative of the worldwide olive diversity (**Supplementary Figure S2**). The 653 genotypes were grouped in two main clusters, where the spontaneous genotypes clustered really close to each other and with the Italian cultivars and only in a few cases with the Greek cultivars (**Supplementary Figure S2**). Moreover, few spontaneous trees are distributed all over the dendrogram, while none of the studied genotypes were present in the clusters of Spanish and Middle Eastern cultivars.

**Figure 2:**
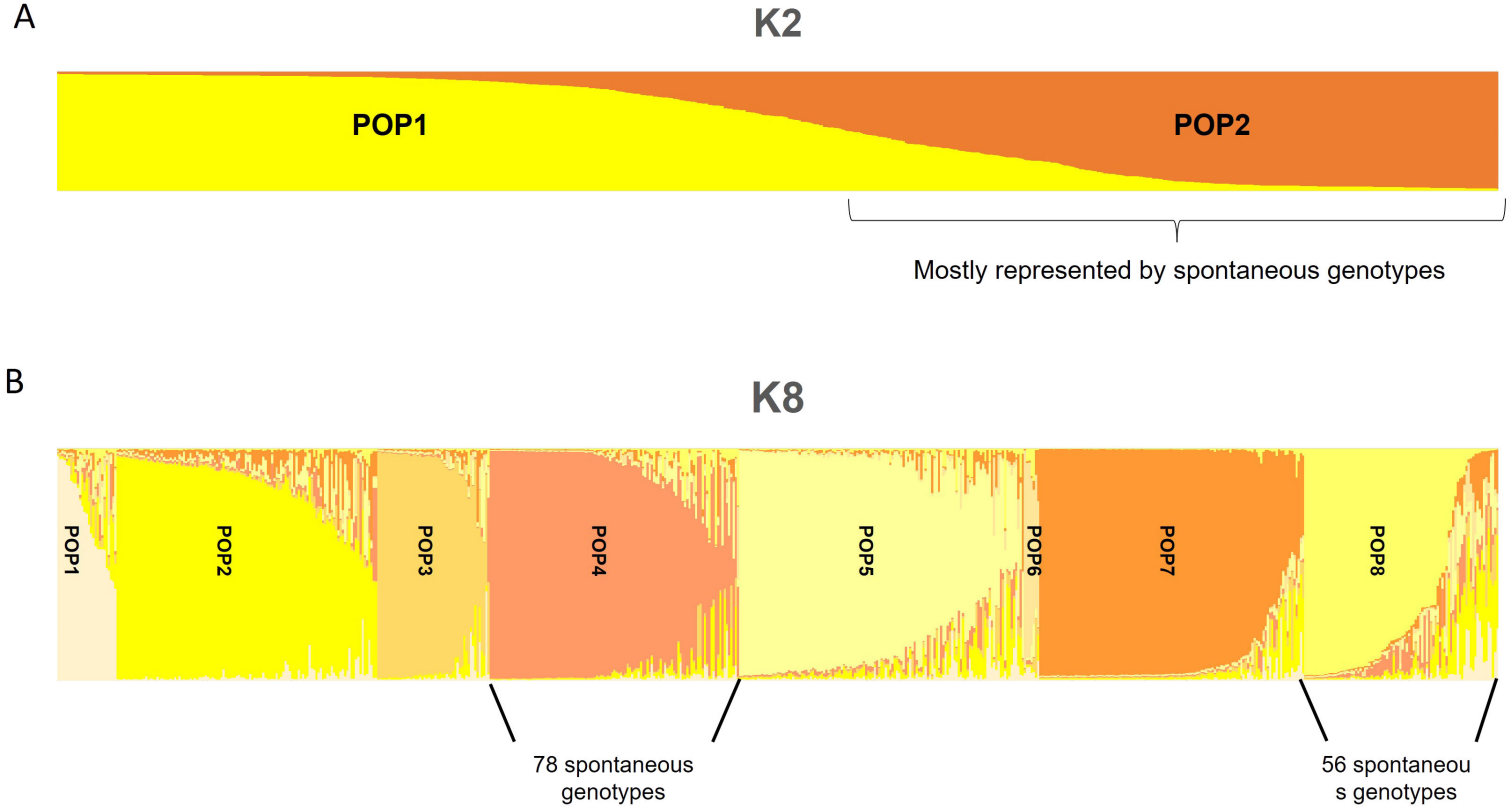
Population structure analysis of data on the 171 unique genotypes and 482 known cultivars. Stabilization, in terms of log-likelihood values of ΔK at K = 2 **(A)** and K = 8 **(B)**, is shown.

The population structure analysis of data on the 171 selected genotypes and 482 internationally known cultivars showed stabilization in terms of log-likelihood values of ΔK at K = 2 at first and, assigning individuals to a population for values above 60%, except the cases of intermixed genotypes (**Figure 2A**). Most genotypes were placed in POP2, while some genotypes, S71_S, S314_R, S12_S, S280_S, S259_T, S168_S, GM10_T, S236_S, S75_S, GM28_T, and S9_S, were assigned among the worldwide genotypes in the first population (POP1). Moreover, some of these such as S177_R, GM24_T, and S278_R had a high intermixed value. The second stabilization point of Bayesian analysis was fixed at K = 8 (**Figure 2B**). In POP1, the cultivars were mostly of East Mediterranean origin and some Italian ones, and only one spontaneous tree was present in this population. The second population (POP2) was represented by a few spontaneous genotypes, both tolerant and susceptible to *Xfp*, together with several Sicilian cultivars, some of the other Italian cultivars from the centre of the Peninsula, and, finally, cultivars from Greece, Turkey, and France. POP3 includes all East Mediterranean and Iranian cultivars, such as Shami, Sourani, Elmacik, Zard, Fishomi, and Mari, from that area; none of the studied genotypes fell into this cluster. Seventy-eight (78) out of 139 spontaneous genotypes were present in POP4, in which the Italian cultivar was Ogliarola salentina, with some of the most diffused cultivars from Tuscany such as Leccino, Americano, Frantoio, Leccio del Corno, and Pendolino. In this POP, three out of seven highly resistant genotypes to *Xfp* were included. A mix of international cultivars such as Canino, Dokkar, Olivastra seggianese, and Koroneiki together with the spontaneous genotypes S175_R, GM21_R, GM32_R, S277_T, S274_R, and S75_S, with most of them resistant to *Xfp*, were assigned to POP5. POP6 was represented by only seven genotypes including three spontaneous genotypes, three cultivars from the eastern side of the Mediterranean Basin, and the cv. Simone autochthonous of Apulia. In POP7, most of the cultivars had Spanish origin, and as already observed in the NJ tree made with 653 olive genotypes, none of the spontaneous plants were genetically linked to the cultivars originating from this country. In POP8, 56 spontaneous genotypes showing a unique genetic profile were present, and the most important cultivars assigned to this POP were Cellina di Nardò, Nociara, and FS-17, with several intermixed cultivars (**Figure 2B**). Based on these results, all genotypes that were not related to known olive varieties were placed in the same genetic population at k = 2 (POP2). Moreover, 134 genotypes out of 139 were placed only in two populations (POP4 and POP8) when K was selected to be equal to 8 (**Figures 2A, B**).

### Paternity analysis

Parentage analysis identified the first and second candidates for 95 genotypes, which are characterized by few trio locus mismatching (from zero to three, from a total of 20 alleles) out of 139 spontaneous trees (**Supplementary Table S3**). For the remaining 41 genotypes, only one candidate parent was identified, while in three cases, S277_T, S203_S, and S50_HS, none of the parents were identified.

**Figure 3:**
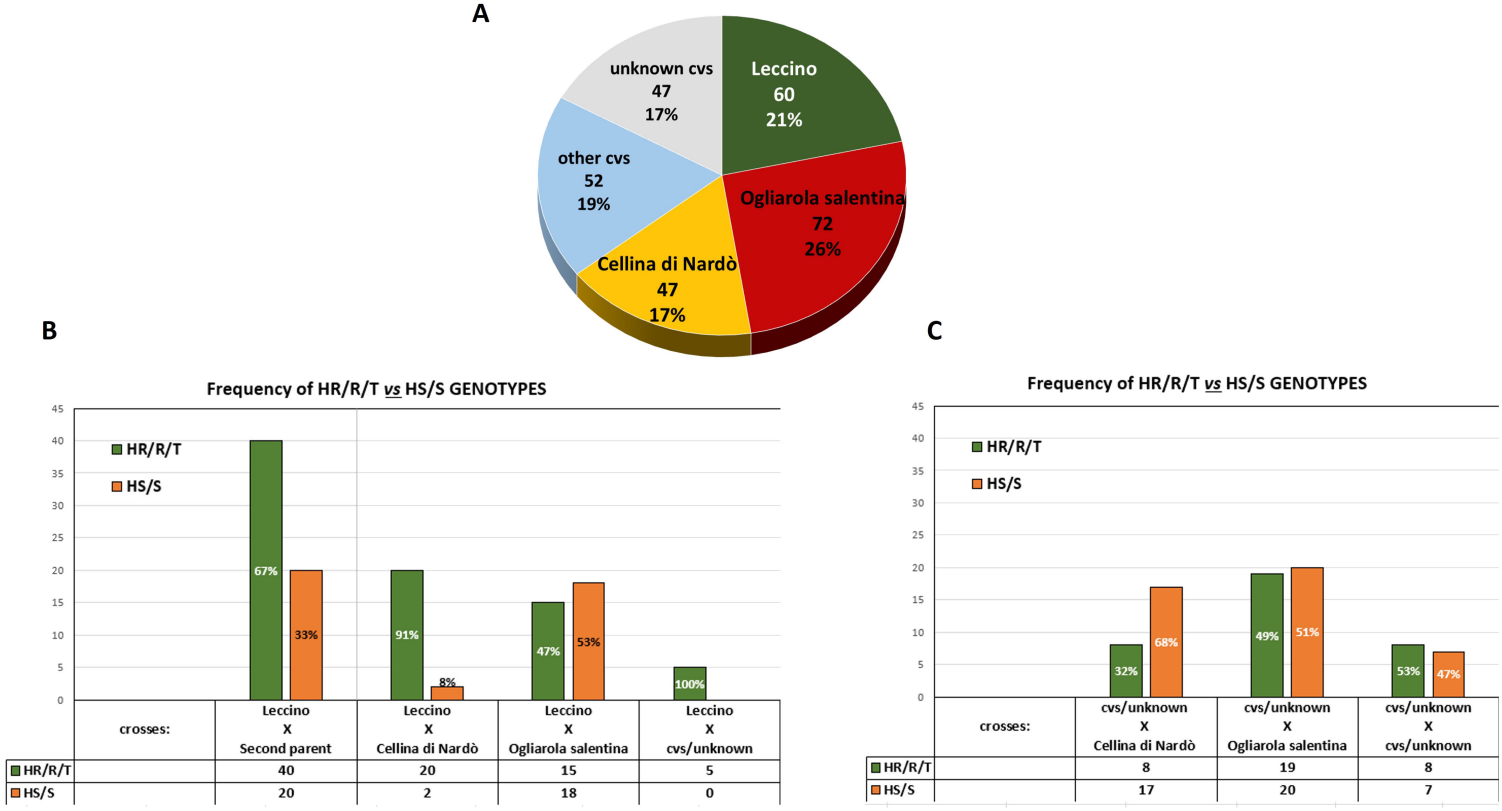
Parentage analysis. **(A)** Pie chart shows the frequency of the cultivars identified in the parental pairs of the 139 seedlings (Ogliarola salentina, Cellina di Nardò, and Leccino). **(B)** Distribution of the phenotypes recorded in the seedlings derived from Leccino. **(C)** Distribution of the phenotypes recorded in the seedlings originating from different cultivars other than Leccino.

The most frequently identified parents belong to the most common cultivars of the area, Ogliarola salentina, Leccino, and Cellina di Nardò, which were identified in the parental pairs of 72, 60, and 47 crosses, respectively (**Figure 3A**). In detail, in 33 out of 60 crosses involving the cv. Leccino, the second parent was cv. Ogliarola salentina, while 22 progenies had cv. Cellina di Nardò as the second parent. The 52 crosses that did not include the three cultivars mentioned above have different cultivars contributing to the parental pairs such as the Apulian varieties Nociara, Nolca, and Silletta, together with other Italian cultivars widely diffused as Cipressino and Frantoio (**Supplementary Table S3**; **Figure 3A**).

### Correlations between phenotypes and parental pairs

Among these 139 genetically unique genotypes when Leccino was one of the parents of the crosses, the majority (67%) of the progeny had an HR/R/T phenotype, while the remaining 33% showed an HS or S phenotype (**Figure 3B**). Conversely, excluding the spontaneous seedlings derived from Leccino, those from Cellina di Nardò and Ogliarola salentina showed, in the majority of the crosses HS or S phenotypes, respectively, 68% and 51% (**Figure 3C**).

Analysis of the phenotypes of the crosses between Leccino and Cellina di Nardò or Leccino and Ogliarola salentina indicates that the two main susceptible cultivars clearly affect the frequency of the genetic traits of resistance in the progenies. More than 90% of crosses between Leccino and Cellina di Nardò express an HR/R/T phenotype, whereas this percentage drops to 47% in the case of the crosses Leccino × Ogliarola salentina. Such outcomes reflect the highest susceptibility to *Xfp* of the cultivar Ogliarola salentina compared to Cellina di Nardò (**Figure 3B**; **Supplementary Table S3**).

### RNA-seq analysis on three selected resistant genotypes

**Figure 4:**
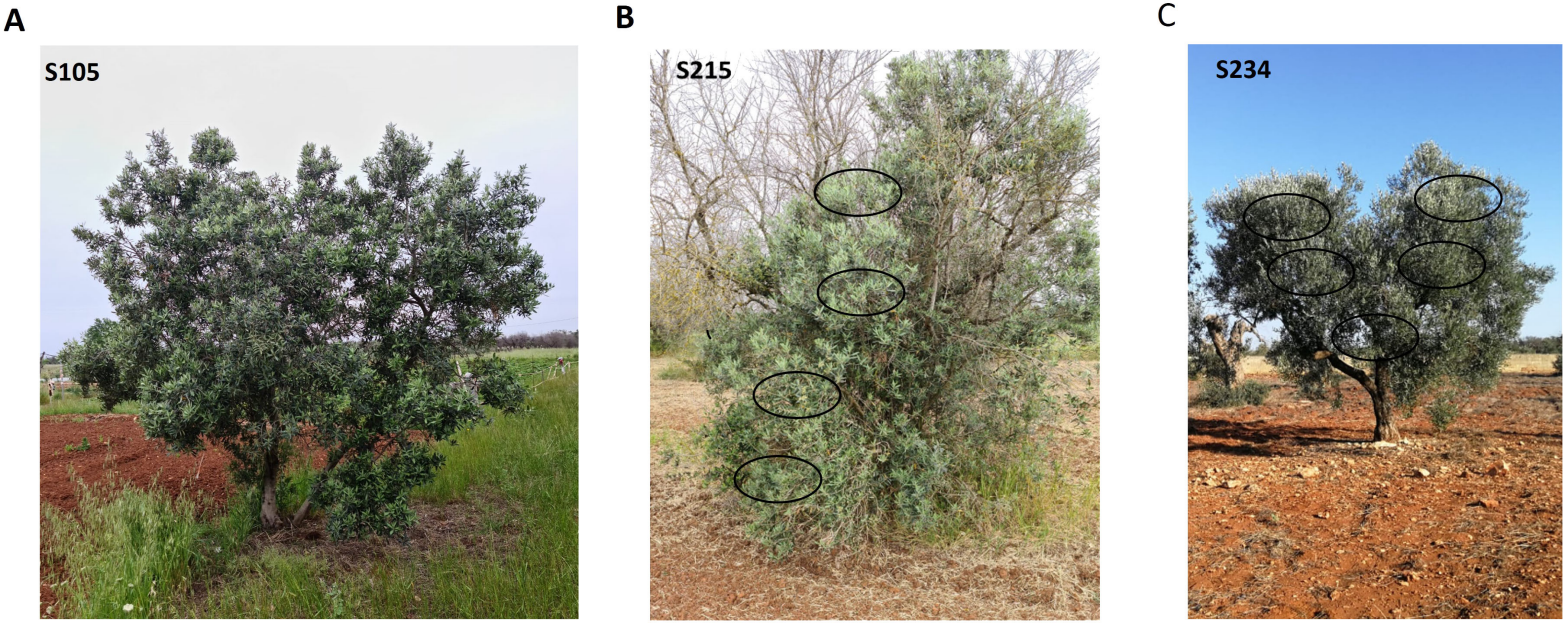
Olive trees of the genotypes selected for transcriptomic analysis. Olive tree S105 **(A)**, S215 **(B)**, and S234 **(C)**. S215 originates from the rootstock of a dead tree of the cultivar Ogliarola salentina. Five subsamples were collected from each tree.

Three spontaneous seedlings resulting from the crosses of Leccino × Cipressino—”S105” and “S215”, respectively classified HR and R, and Leccino × Ogliarola salentina, S234, classified R—were selected for the RNA-seq analysis (**Figure 4**). Quantitative PCR showed that *Xfp* was found erratically distributed in the canopy of these three olive seedlings. Total RNA extraction and construction of libraries were carried out by collecting multiple shoots (**Figure 4**; **Table 2**). A total of 704,768,698 100-bp paired-end (PE) reads were generated through the Illumina platform from 15 samples (5 samples × 3 selected olive genotypes) (**Figure 4**; **Table 2**). The reads had a high quality with average phred scores Q20 and Q33 of 98.5 and 95.5, respectively. Reads were mapped to the cv. Farga genome version 9 (Julca et al., 2020) (**Table 2**).

**Table 2:** *Xfp* detection and RNA-seq data on tissues from three selected spontaneous seedlings.

Olive tree	Sample ID	qPCR results: Cq value and estimated CFU/mL	Total number of raw reads	Number of reads (mapping rate %) cv. Farga_OEA9	Condition/cluster PCA
**S105**	**S105_N1_5**	0	51,738,948	41,787,669 (80.8)	**Xf_neg**
	**S105_N3_7**	0	43,146,068	28,604,720 (66.3)	
	**S105_P4_4**	27.56 (1.68E+04)	53,206,518	44,044,670 (82.8)	**Xf_DDpos**
	**S105_1_8**	33.60 (2.56E+02)	50,679,566	38,281,320 (75.5)	
	**S105_3_7**	29.86 (3.42E+03)	45,321,692	34,232,245 (75.5)	
**S215**	**S215_N2_12**	0 (*)	39,814,216	33,678,065 (84.6)	**Xf_DDpos**
	**S215_P1_8**	24.66 (1.26E+05)	48,750,452	40,017,090 (82.1)	
	**S215_15_15**	0	46,628,346	34,949,963 (75.0)	**Xf_neg**
	**S215_17_16**	0	40,566,968	32,787,093 (80.8)	
	**S215_9_14**	0	43,468,698	34,853,671 (80.2)	
**S234**	**S234_N2_18**	0 (*)	55,856,138	47,643,756 (85.3)	**Xf_DDpos**
	**S234_P3_16**	29.04 (6.04E+03)	40,782,520	34,781,583 (85.3)	
	**S234_13_22**	0	45,189,426	36,722,134 (81.3)	**Xf_neg**
	**S234_18_21**	0	49,976,950	39,290,416 (78.6)	
	**S234_8_20**	0	49,642,192	40,377,123 (81.3)	

Quantitation cycle values (Cq) generated by qPCR and basic statistics of samples subjected to RNA-seq. The number of raw reads and relative mapping rate on the cv. Farga genome are reported. The column/cluster PCA indicates the Xfp status as defined based on the principal component analysis. Xf_neg = Xfp negative samples; Xf_DDpos = Xfp-positive samples. (*) Samples with Cq value of “0” but clustering with infected samples (Xf_DDpos group).

**Figure 5:**
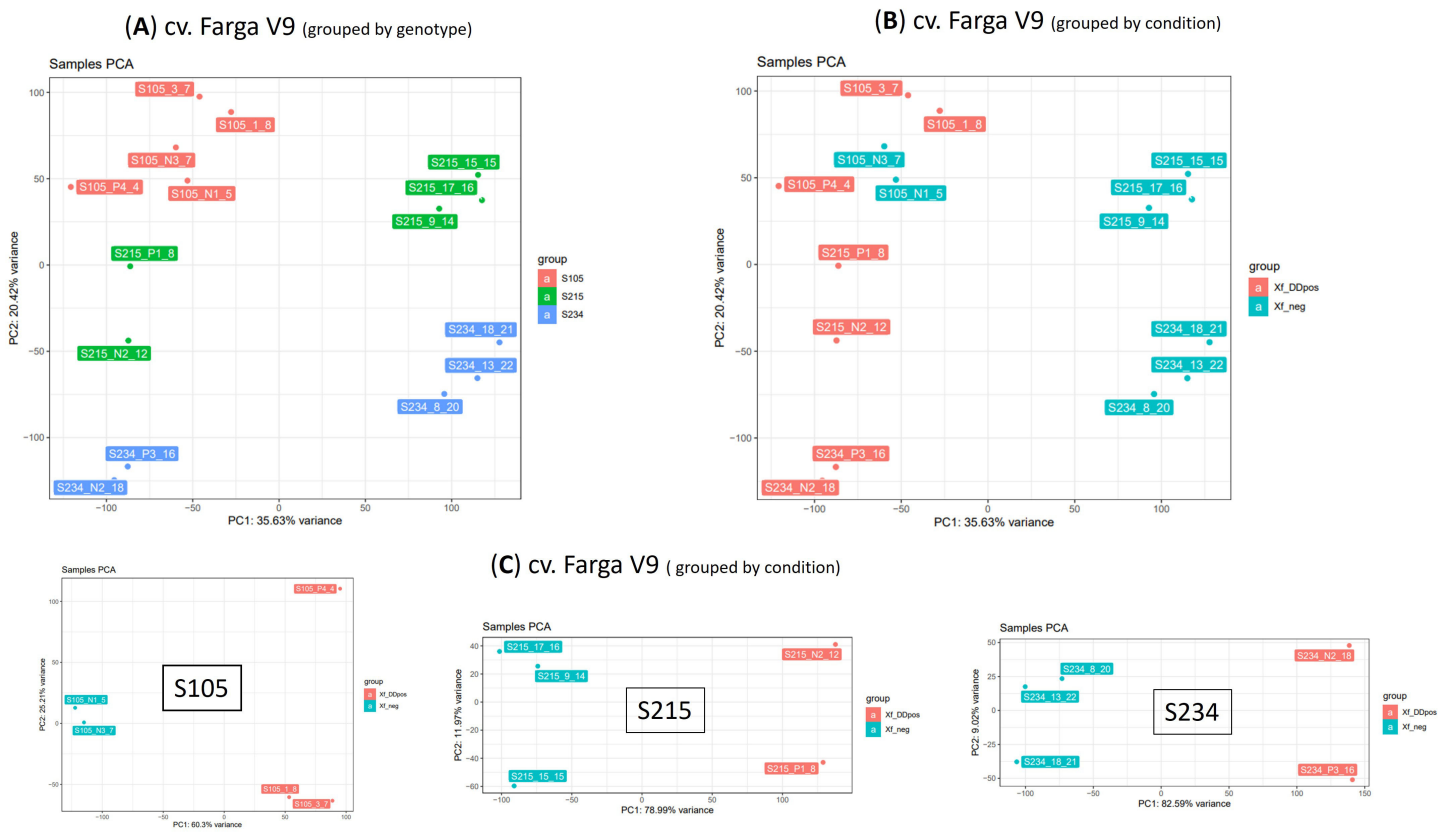
Principal component analysis (PCA) on 15 RNA-seq samples. PCA was performed on dataset of normalized counts from mapping RNA-seq libraries on cv. Farga genome; samples were grouped by genotype **(A)** or condition **(B)** or analysed per genotype **(C)**.

Principal component analysis (PCA) revealed that the RNA-seq data have sufficient structure to distinguish samples according to the genotype (20.42% of variance PC2 component) (**Figure 5A**) or condition (35.6%) (**Figure 5B**). This analysis also suggested a distinction of samples according to the PC1, whose variance explains the clear separation of genotypes S215 and S234. We therefore investigated the PCA grouping of libraries from the three individual genotypes (**Figure 5C**). This representation showed that the PC1 component explains most of the variance and clearly separates the libraries into two groups (**Figure 5C**). In contrast, this clustering fits with the infection condition for the S105 genotype since it separates samples according to the real-time quantitative PCR (qPCR) results; this was not completely in line with that of the S215 and S234 genotypes. For example, S215_N2_12 and S234_N2_18, both testing negative to *Xfp* by qPCR assay (Xf_neg), clearly clustered (PC1 supported by 77.74% and 72.85% of the variance, respectively) with Xf_DDpos samples (**Table 2**). However, considering that tissues originate from asymptomatic plants that have low bacterium load, the negative qPCR results could be affected by the detection limit of qPCR assay. Thus, for the gene expression analyses, samples were categorized according to the PCA separation rather than the qPCR results: positive samples “Xf_DDpos” (S105_P4_4, S105_1_8, S105_3_7, S215_N2_12, S215_P1_8, S234_P3_16, and 234_N2_18) or negative “Xf_neg” (S105_N1_5, S105_N3_7, S215_9_14, s215_15_15, S215_17_16, S234_8_20, S234_13_22, and S234_18_21 (**Table 2**; **Figure 5C**). This clustering was also confirmed by a sample-to-sample distance analysis by correlation distance method on the RNA-seq data mapped on the Farga genome, as it grouped samples according to the genotype and Xf_DDpos/Xf_neg condition (**Supplementary Figure S3**).

**Figure 6:**
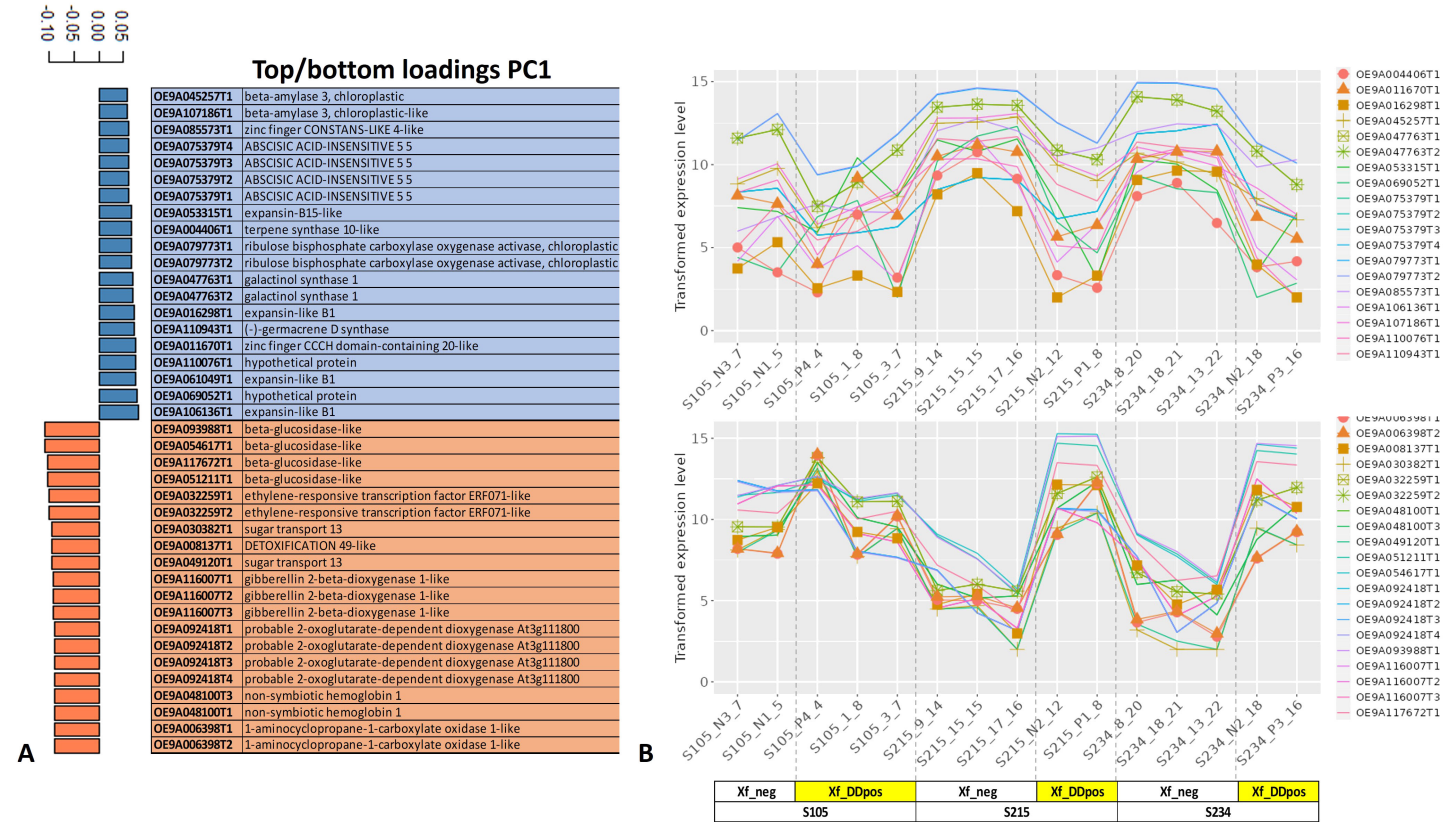
Top/bottom PC1 loading genes. The first 20 top (blue, downregulated) and bottom (orange, upregulated) loading genes directing the separation of the samples according to the PC1 are reported **(A)**, and related transformed expression values are shown for each sample in the line graph **(B)**.

Major genes driving the separation of samples Xf_DDpos and Xf_neg according to PC1 showed that Expansin-like B1 (OE9A106136T1, OE9A061049T1, and OE9A016298T1), galactinol synthase (OE9A047763T1,2), ribulose bisphosphate carboxylase oxygenase activase (OE9A079773T1,2), beta-amylase 3, chloroplastic (OE9A045257T1 and OE9A107186T1), and ABSCISIC ACID-INSENSITIVE 5 (OE9A075379T1,2,3,4) are among the 20 top loading genes whose transcripts are downregulated in Xf_DDpos plants according to the transformed expression level graph. In contrast, beta-glucosidase-like proteins (OE9A093988T1, OE9A054617T1, OE9A117672T1, and OE9A051211T1), gibberellin 2-beta-dioxygenase 1-like (OE9A116007T1,2,3), sugar transport protein 13 (OE9A030382T1 and OE9A049120T1), probable 2-oxoglutarate-dependent dioxygenase At3g11180 (OE9A092418T1,2,3) and ethylene-responsive transcription factor ERF071-like (OE9A032259T1,2) were among the 20 bottom loading genes mainly upregulated in the Xf_DDpos 215 and 234 genotypes, as shown in the relative transformed expression level graph (**Figures 6A, B**).

### Identification of differentially expressed genes

**Figure 7:**
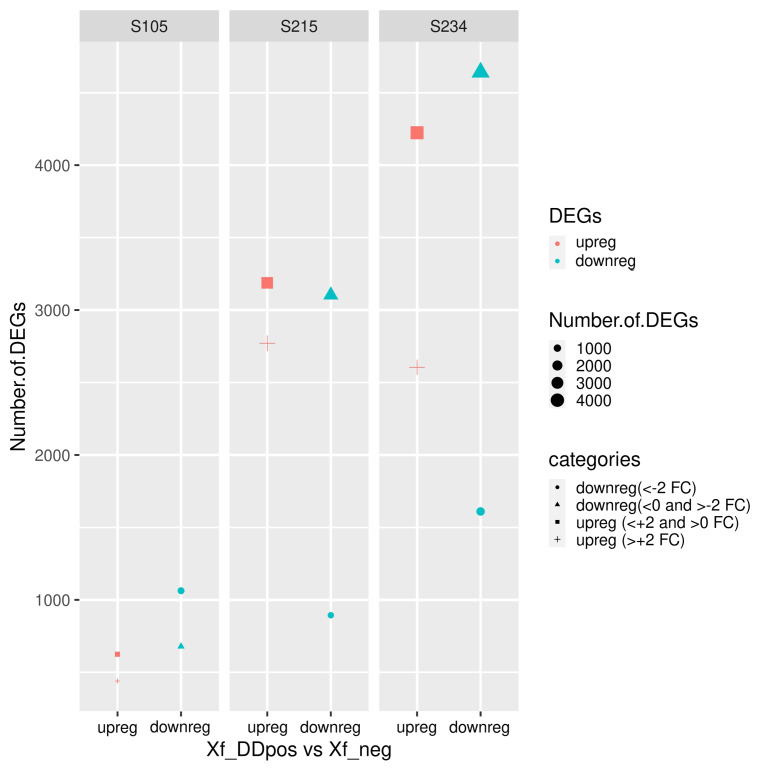
Statistics of differentially expressed genes (DEGs) in the three genotypes. DEGs are differentiated according to the decreased log2 fold change (FC). Upregulated (upreg) and downregulated (downreg) genes were reported in red and blue colours, respectively; points were sized according to the variable (“number.of.DEG”); different shapes in the plot indicate the four categories according to the value of FC: upreg (>+2 FC), upreg (<+2 and >0 FC), downreg (<0 and >−2 FC), and downreg (<−2 FC).

The exploratory analysis of the RNA-seq data indicates that the PC1 component, which explains most of the variance, led to a clear distinction of Xf_DDpos/neg samples, likely suggesting that a localized response to *Xfp* occurs in colonized sectors of the tree canopy. We therefore set this clustering (**Table 2**) as a condition for the successive examinations. The DESeq2 analysis on the datasets obtained by mapping reads on cv. Farga v9 genome indicated that the “S234” genotype strongly perceived the pathogen presence showing the highest number of differentially expressed genes (DEGs) [p < 0.001 = 17,227, filtered by false discovery rate (FDR) <0.05 = 13,080], compared to those of the “S215” genotype (p < 0.001 = 13,030 filtered by FDR < 0.05 = 9,956) and those of the “S105” genotype DEGs (p < 0.001 = 4,513 filtered by FDR < 0.05 = 2,897) (**Figure 7**). This DEG distribution fits with data arising from the phenotyping since genotype S105 ranked “highly resistant”, which is clearly distinct from the other two seedlings classified “resistant”. DEGs having a lesser fold change (FC) ≥2 and ≤−2 will form the dataset for the subsequent functional enrichment study aiming to investigate the metabolic pathways affected by *Xfp* infection by MapMan4 analysis.

### MapMan metabolic pathway analysis of DEGs

A total of 17,227, 13,030, and 4,513 DEGs for each genotype were mapped to 1,972 hierarchically organized functional categories (BINs). To have a better overview of biotic stress-related pathways, new customized ontology-derived pictorial representations of BINs were created in MapMan4.

**Figure 8:**
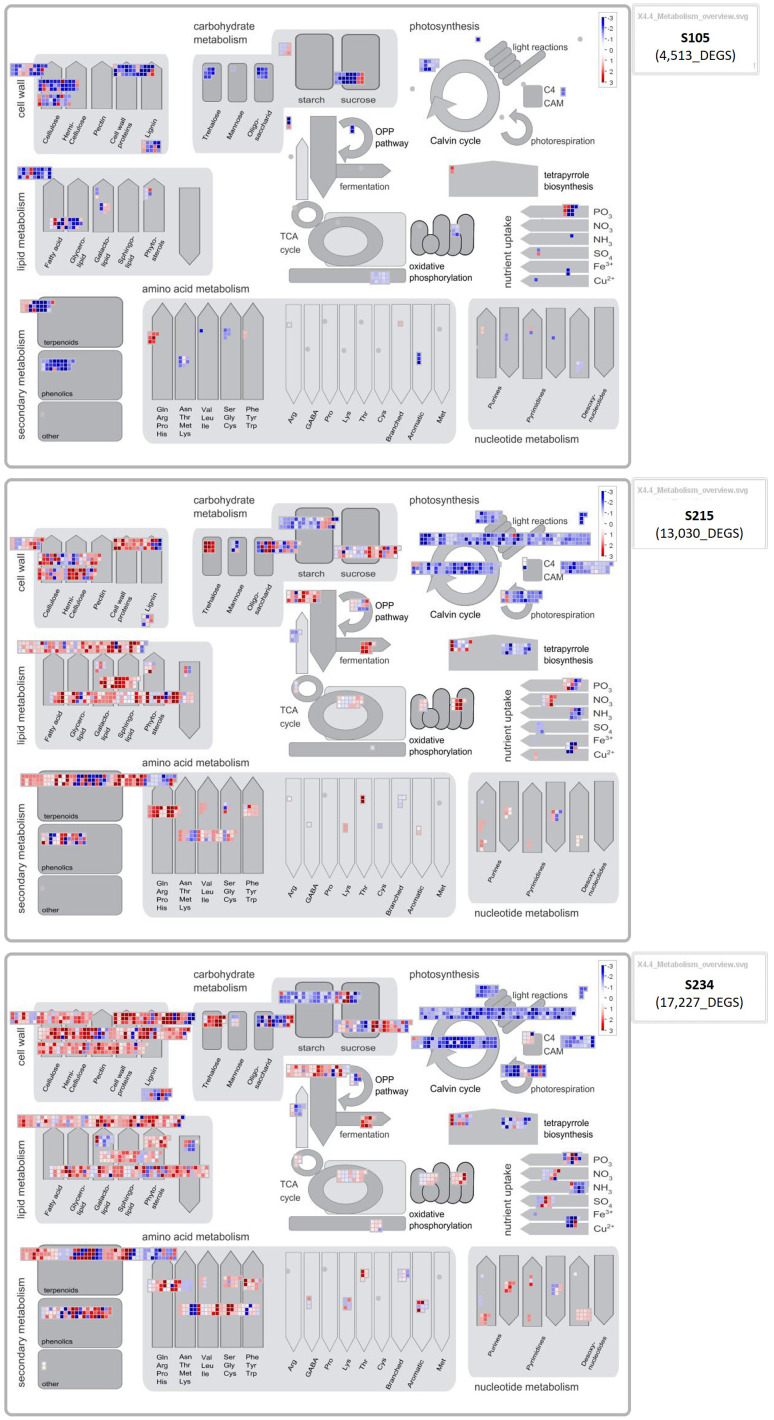
MapMan metabolism overview showing the differentially expressed genes (DEGs) in each genotype (S105, S215, and S234). Results obtained from DESeq2 were loaded into the MapMan Image Annotator module to generate the metabolism overview map. The different colours represent the decreased log2 fold-change values of the gene expression levels in response to Xf infection: blue represents downregulated DEGs, and red represents upregulated DEGs.

The BIN representing the “metabolism overview” clearly showed that the higher number of DEGs that were downregulated in the S234 and S215 genotypes were related to the photosynthesis process (**Figure 8**). A detailed BIN representation (**Supplementary Figure S4**) shows that DEGs having the lowest fold change (<−5) were transcripts coding for chlorophyll *a*–*b* binding domain-containing proteins (OE9A075888T1 and OE9A096707T1), which enter the composition of the photosystem II. DEGs upregulated in the S234 and S215 genotypes belong to functional categories related to “cell wall”, “lipid metabolism”, and “secondary metabolism” (**Figure 8**).

Within the “phenolics” subcategory of “secondary metabolism”, we found transcripts coding for DOWNY MILDEW RESISTANCE 6-like (DMR6-like, OE9A054976T1, and OE9A079697T1 similar to VvDMR6.2/1), while in the “cell wall, pectin” subcategory, DEGs were represented by genes coding for pectin methyl esterase inhibitor family protein (OE9A103328 and OE9A018498). Moreover, upregulated DEGs in S234 and S215 were those annotated as “cell wall, expansin activities” code for alpha-class expansin, Expansin A-1 (OE9A055620), and for beta-class expansin, Expansin-like B1 (OE9A100033), respectively. Among the BINs related to the plant–pathogen interactions, those representing the “external stimuli response”, subcategories “light” (abiotic stress) and “pathogen” (biotic stress) showed a higher number of DEGs in S234 (“abiotic” 144 and “biotic” 105) and S215 (“abiotic” 113 and “biotic” 98) genotypes compared to the S105 (“abiotic” 39 and “biotic” 33) (**Supplementary Figure S5**). Interestingly, all DEGs related to “pathogen” were upregulated in S234 and S215, with the highest values of fold expression for genes coding for a polygalacturonase inhibitor-like protein (OE9A070676T1 and OE9A036088T1) and ACBP60 family proteins similar to protein SAR DEFICIENT 1 (OE9A093289T1 and OE9A068952). Moreover, transcripts in the response to the “toxic” subcategory were found downregulated in S105 and upregulated in the S234 and S215 genotypes (**Supplementary Figure S5**). A BIN representation of receptor-like kinases (RLKs), key components of the signalling pathway including pathogen recognition, was created according to Shiu and Bleecker (2001). This functional clustering was composed of several DEGs annotated as WAK-like, DUF26, S-Domain1,2,3 subfamilies of RLKs and DEGs annotated as RLCK-VII, which were more upregulated in the S234 and S215 genotypes, while DEGs of the LRR XII subfamily, coding for probable LRR receptor-like serine threonine-kinase At3g47580 (OE9A013998T1 and OE9A100116T1), were, in addition, highly expressed in the S105 genotype (**Supplementary Figure S6**). Moreover, a higher number of Receptor-like cytoplasmic kinases (RLCKs), annotated as PBS1, was upregulated in S234 and S215 than in S105. Analysis of DEGs annotated in BINs of regulatory processes such as transcription factors (TFs) (**Supplementary Figure S7**), currently distinguished in 91 families by MapMan4, showed that a higher number of WRKY, NAC, and ERF/DREB/AP2 transcripts, known to be involved in biotic stress responses, were upregulated in XfDD samples of the S215 and S235 genotypes compared to those of S105. Among the most upregulated classes of TFs (decreased fold change >+5), a WRKY transcription factor 30 (OE9A105450T1 and OE9A042006T1), a NAC domain-containing 2-like (OE9A057226T1 and OE9A102768T1), and ethylene-responsive transcription factors ERF071-like (OE9A032259T1) and ABR1-like (OE9A066785T2) were found.

### Gene ontology enrichment analysis of DEGs

The Gene Ontology (GO) enrichment analysis of the 440 upregulated DEGs in the S105 genotype identifies “protein serine/threonine kinase activity” (GO:0004674), “ADP binding” (GO:0043531), and “protein kinase (GO:0004672) molecular functions (MFs) having higher FDR enrichment values (2.40E−08 and 2.30E−13) (**Supplementary Table S5**). A careful examination of the enriched transcripts in these three categories discloses the presence of receptor-like kinases (probable LRR receptor-like serine threonine-kinase At3g47570, leucine-rich repeat receptor-like serine threonine tyrosine-kinase SOBIR1, and wall-associated receptor kinase 2-like, L-type lectin-domain containing receptor kinase-like) and several disease resistance proteins (late blight resistance homolog R1A-10, with disease resistance RGA3 and RPM1-like among the DEGs having the highest decreased fold change) (**Supplementary Table S4**). In contrast, differing from that of S105, molecular functions enriched from upregulated DEGs of genotypes S215 and S234 (**Supplementary Tables S7**, **S9**) have more commonalities, as they are referred to as “Transcription factor activity, sequence-specific DNA binding” (GO:0003700), “Heme binding” (GO:0020037), “Sequence-specific DNA binding” (GO:0043565), and Calcium ion binding” (GO:0005509), which further marks the different responses to the infections of these two seedlings compared with S105. Indeed “ADP binding” (GO:0043531), “Protein serine/threonine kinase activity” (GO:0004674), and Protein kinase activity” (GO:0004672) are the highest enriched categories in this genotype. Several transcription factors involved in the ABA signalling pathway, plant development or defence response, such as ethylene-responsive transcription factors ERF071-like (OE9A032259) (Yelli et al., 2018) and ERF096-like (OE9A102306) (Catinot et al., 2015), and probable WRKY transcription factor 53 (OE9A042006) and 40 (OE9A054922) were in the GO:0003700 (Transcription factor activity, sequence-specific DNA binding) and GO:0043565 (Sequence-specific DNA binding) terms, which were enriched in S234 and S215 (**Supplementary Tables S6**, **S8**). Moreover, a cationic peroxidase 1-like (OE9A024386) and other peroxidases are enriched in genotype S234. These secreted class III peroxidases belong to the class 9 subfamily of pathogenesis-related proteins (Almagro et al., 2009). GO analysis of the downregulated genes shows that the “Nucleosome” (GO:0000786) cellular component is the most supported term in genotype 105 (**Supplementary Table S11**), being enriched of several members of the core histone complex (histone H2B, H4, and H3) (**Supplementary Table S10**). Many chlorophyll *a*–*b* binding proteins are downregulated in genotype S215 (**Supplementary Table S12**) and contribute to enriching the terms “Chloroplast thylakoid membrane” (GO:0009535) and “Chlorophyll binding” (GO:0016168) (**Supplementary Table S13**). These apoproteins enter the composition of the light-harvesting complex of photosystem II (PSII). The expression of these proteins is repressed by the ABA-responsive WRKY transcription factor 40 (Liu et al., 2013), which, indeed, is found upregulated in this genotype as reported above.

“Carbon utilization” (GO:0015976) is the biological process enriched in downregulated genes in genotype 234 (**Supplementary Table S15**), among which a key enzyme of the Calvin cycle, a phosphoribulokinase, chloroplastic-like that regenerates d-ribulose-1,5-bisphosphate, is downregulated (**Supplementary Table S14**).

### *Xfp* screening test on candidate new resistant genotypes

**Figure 9:**
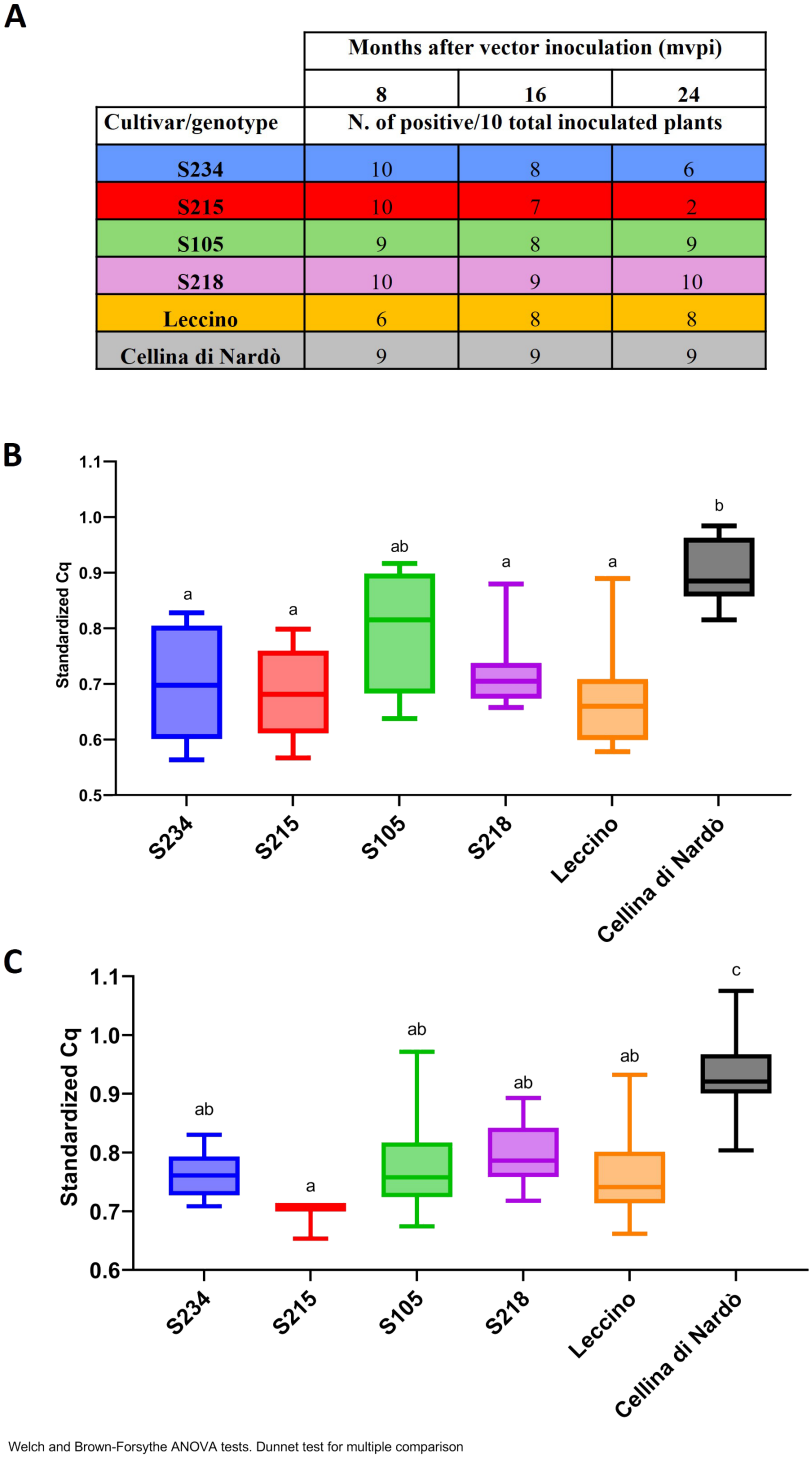
Quantification of Xf population by qPCR in the *Xfp*-inoculated olive genotypes. **(A)** Table with the number of positive plants recorded at 8, 16, and 24 mpvi for each genotype and for the controls (Leccino and Cellina di Nardò). **(B, C)** qPCR standardized values obtained with the following formula = 1/((Cq rimM)/(Cq COX)) for the positive plants at 16 mpvi and 24 mpvi, respectively. Data were subjected to ANOVA, and the means of treatments were compared using Dunnett’s test (p ≤ 0.05). Bars marked with the same letter are not statistically different.

During the vector transmission test carried out in September 2021, individual replicates for each genotype were tested 8 months post-vector inoculation (mpvi) by collecting mature leaves. The results of the diagnostic tests showed high efficiency of transmission. All replicates tested positive, except for the control Leccino, for which four out of 10 replicates tested negative, and for Cellina di Nardò and S105, in which one replicate escaped the infection (**Figure 9A**).

When diagnostic tests were repeated 16 mpvi using the xylem tissues recovered from the shoots, the results showed that albeit the bacterium was efficiently transmitted in the leaves where the insects were forced to feed, the systemic colonization of the plants did not occur in all replicates. For the genotypes under testing, the number of infected plants ranged from 7 to 9 out of 10 replicates. Similarly, in Leccino, eight replicates out of 10 turned out to be positive. In the case of Cellina di Nardò, all nine plants initially testing positive were confirmed to be systemically infected, with one replicate remaining negative (most likely because the transmission failed) (**Figure 9A**). Although in the genotypes under testing the estimated bacterial population varied among the replicates, their median sizes were significantly lower than those recorded in the susceptible replicates of Cellina di Nardò, with the only exception of S105 (**Figure 9B**). As expected, the occurrence of a high level of bacterial population in the control plants of Cellina di Nardò resulted in the appearance of the typical symptoms of shoot dieback and desiccation. The initial sign of *Xfp*-induced dieback was observed in one replicate at 12 mpvi. These progressed rapidly, with eight out of nine infected replicates of Cellina di Nardò showing symptoms at 16 mpvi, two of which developed severe desiccation with the plants that completed died (**Supplementary Figure S8A**). At the same time, none of the Leccino-infected replicates showed withering or desiccation phenomenon, and for the genotypes under test, these were recorded to a limited extent only in a few replicates. More specifically, such alterations were recorded in two replicates of genotype S218 (Leccino × Cellina di Nardò) and two replicates of genotype S234 (Leccino × Ogliarola salentina) (**Supplementary Figure S8C**). None of the infected plants of the remaining two genotypes (S105 and S215), both derived from the cross Leccino × Cipressino, showed alterations resembling those previously reported.

At 24 mpvi, the difference in genotype response to *Xfp* became much more evident. All the infected replicates of Cellina di Nardò showed severe desiccations, resulting in the complete collapse of the plants (**Supplementary Figure S9B**); replicates of Leccino S218 and S234 (**Supplementary Figures S9A, D, F**) showed mild symptoms consisting in shoot defoliation and dieback, while the plants of genotypes S105 and S215 did not show any desiccation phenomenon (**Supplementary Figures S9B ,C**). Moreover, the estimation of the *Xfp* population sizes showed that those from the genotypes are significantly lower than those of Cellina di Nardò and more similar to those of Leccino (**Figure 9C**). Overall, under our experimental conditions, all Leccino-bearing genotypes showed resistance against the bacterial infection, thus confirming the field phenotype. This response was similar to that of the parental Leccino in S218 and S234 and much higher than that in genotypes S105 and S215. Interestingly, while bacterial host colonization did not vary in time in the replicates of S105, S215 efficiently counteracted the bacterial plant colonization, with only two replicates testing positive at 24 mpvi (**Figure 9A**).

## Discussion

Because of its long life and productive cycle, olives must cope with several biotic stresses, i.e., insects, fungi, bacteria, and viruses, and the understanding of the molecular basis of the olive response to these stressors is of fundamental importance for breeding programs towards sustainable and innovative crop management solutions (Arias-Calderón et al., 2015; Cabanás et al., 2015; de Pascali et al., 2019). The challenges posed by the recent introduction in the Mediterranean olive-growing area of the harmful bacterium *X. fastidiosa* and the expansion of this crop in new areas where the bacterium is already established (i.e., south and north America) make it necessary to explore the high variability of the *Olea* species for characterizing the genetic determinants controlling the plant response to this biotic stress. The present study is the first to exploit the genetic diversity of spontaneous olive seedlings that survived the detrimental impact of *Xfp* infections in the Apulia region, where one of the most severe worldwide *Xylella* epidemics emerged in the last decade. Although the data were mainly collected under field conditions, with many variables affecting host responses to the infections and mainly referred to observations on a single individual tree per genotype, the long-term period of the field selection proved to be effective in differentiating genotypes based on their susceptibility and ultimately to identify potential new sources of resistance to *Xfp*.

Investigations on these populations can contribute to rapidly advance the characterization of the traits of resistance observed in this species. The phenotypic response to *Xfp* of more than 170 spontaneous genotypes was studied during 6 years of field observations and laboratory tests, and their genetic relatedness with 480 national and international olive cultivars was analysed. In addition, to dissect the olive interactions with *Xfp* and to facilitate the identification of candidate genes contributing to the resistance phenotype, the transcriptomic profiles of three genotypes were deeply analysed.

### Genetic differences between the spontaneous genotypes

The Bayesian analysis of population differentiation revealed the clear separation of spontaneous genotypes mostly in two and eight POPs. Cultivars from two of the most important olive-growing countries, such as Spain and Greece, did not show any genetic similarity to the analysed spontaneous olive trees. In contrast, 52% of HR, R, and T genotypes were present in the POP4, which includes the susceptible Ogliarola salentina and the resistant cultivar Leccino, together with other highly diffused varieties, Frantoio and Pendolino. Several HR, R, and T genotypes were assigned also to POP8, which they grouped with the susceptible cv. Cellina di Nardò, the resistant FS17, and the Apulian varieties Nociara, which contributed as parents of some genotypes here analysed. Our results show that the SSR markers are not informative enough to support correlation studies between the genetic clusters and predictive *Xfp* phenotype responses, most likely because SSRs were developed to discriminate olive varieties by selecting the most polymorphic traits and because multiple traits contribute to resistance. However, it was interesting to associate two SSRs, GAPU103a and UDO99-043, with a positive fixation index, which were observed only in the susceptible genotypes, highlighting the possibility that the susceptible cultivars are genetically similar in some parts of their genome. Further analyses such as quantitative trait loci (QTLs) and genome-wide association studies (GWASs) on a large set of olive genotypes both resistant and susceptible could contribute to identifying markers linked to the susceptibility to *Xfp* close to the two mentioned SSRs, confirming this preliminary correlation.

### Cultivar Leccino in the parentage analysis

The parentage analysis clearly assigns the cultivar Leccino as the direct parent in several spontaneous genotypes that did not succumb to *Xfp* infections, suggesting that the genetic heritage of this cultivar can be exploited to disclose the mechanisms of resistance and in breeding programs to confer resistance to the progenies.

The analysis demonstrates that Leccino has transferred with high frequency the traits of resistance to *Xfp* to the surviving seedlings, even with the two autochthonous susceptible cultivars, Cellina di Nardò and Ogliarola salentina, a finding that raises hope to preserve the characteristics of these local cultivars in the landscape and agricultural regeneration programs for this devastated area. In contrast, this study also confirms that multiple traits concur to confer resistance to *Xfp* in olive, as highlighted in previous studies targeting anatomical, physiological, and molecular features in resistant vs. susceptible olive trees (Giampetruzzi et al., 2016; Montilon et al., 2022; Surano et al., 2022).

### Plant response to *Xfp* at transcriptomic level

The genome resources of the wild olive (*O. europaea* var. *sylvestris*) and the cultivars Picual and Farga are publicly available (Cruz et al., 2016; Unver et al., 2017; Jiménez-Ruiz et al., 2020). To map the RNA-seq transcripts, we selected version 9 of the cv. Farga because its genome assembly was improved by anchoring it to a genetic map (Julca et al., 2020), and it provides the most comprehensive set of functional annotated genes at the time this work was performed.

Transcriptomic studies enabled to identify large sets of transcripts involved in important metabolic pathways and the mechanisms of stress tolerance. The transcriptomic profiles allowed to identify differentially expressed genes involved in the tolerance to biotic (Leyva-Pérez et al., 2018; Grasso et al., 2017) and abiotic (Bazakos et al., 2015; Guerra et al., 2015; Mousavi et al., 2019, 2022; Karamatlou et al., 2023) stresses.

Regarding the olive response to *Xfp*, Giampetruzzi et al. (2016) firstly described the transcriptomic profiles of field-grown plants of ‘Ogliarola salentina’ and Leccino, showing that the resistant ‘Leccino’ harbour structural genes and/or regulatory elements that counteract *Xfp* infection. In-depth molecular investigations by RNA-seq analysis on three candidate new resistant genotypes showed that genes responding to biotic and abiotic stresses and controlling plant hormone levels to regulate the tradeoff between defence and growth have altered expression upon *Xfp* infection. Indeed, the activation of defence genes subtracts energy from plant growth, a delicate balance between the processes that should be finely tuned by regulating plant hormone levels. Among genes regulating hormone levels, the At3g11180 orthologue encoding 2-oxoglutarate-dependent dioxygenase (JOX1), a 2OG oxygenase that inactivates the plant hormone jasmonic acid (JA) by hydroxylation, was found to be upregulated (Caarls et al., 2017).

This gene is induced in *Arabidopsis thaliana* upon infection with the necrotrophic fungus *Botrytis cinerea* and with infestation by the caterpillar *Mamestra brassicae*, and its knock-out inhibits the root and shoot growth in favour of enhanced resistance to these pathogens and pests. JOX1 finely tunes the JA hormone levels, which need to be controlled to counterbalance the response to biotic stress with the plant growth, a mechanism that is exerted by another oxygenase found differentially expressed, the DOWNY MILDEW RESISTANCE 6-like oxygenase (DMR6-like). DMR6-like was among the upregulated genes annotated in the “secondary metabolism” BIN by MapMan4. It controls the levels of the plant hormone salicylic acid (SA) in *A. thaliana* (Zeilmaker et al., 2015), and its knock-out mutation makes this herbaceous host resistant to the downy mildew *Hyaloperonospora arabidopsidis*, the bacterium *Pseudomonas syringae*, and the oomycete *Phytophthora capsici*. Similarly to JOX1, DMR6-like suppresses plant immunity as their mutation enhances the expression of defence genes. Both are therefore “susceptibility genes” (Koseoglou et al., 2022) whose possible biotechnological application has been demonstrated in grapevine, tomato, potato, and banana (de Toledo Thomazella et al., 2021; Kieu et al., 2021; Tripathi et al., 2021; Giacomelli et al., 2022; Pirrello et al., 2022).

The study of DEGs within each tree clearly indicates that a plant defence response occurs in the tissues invaded by the bacterium, with DEGs being much higher in S215 and S234 than in S105, as also reflected in the associated Gene Ontology studies. In the highly resistant genotype, S105 *Xfp* induces the lowest perturbation of gene expression. The Gene Ontology analysis shows that molecular functions relying on the activity of cell wall-associated protein kinases belonging to the basal immunity response are boosted in *Xfp*-infected tissues. Several RLKs, described in our previous study (Giampetruzzi et al., 2016) or other transcriptomic analyses in citrus (Rodrigues et al., 2013) and grapevine (Choi et al., 2013), were found upregulated in S215 and S234 (WAK-like, DUF26, S-Domain1,2,3, and RLCK-VII) or S105 (LRR-RLK At3g47580). Moreover, NBS-LRR resistance proteins, late blight resistance homolog R1A-10, disease resistance RGA3, and disease resistance RPM1-like are found overexpressed in spontaneous genotype S105. Of note is the upregulation of SOBIR1 in S105, a regulatory LRR-RLK that provides the intracellular kinase domains to LRR-RLPs to allow the cytoplasmic transduction of RLP cell wall-detected stimuli (Wei et al., 2022). Considering that RLK and RLP genes are present in the PdR1b locus of Pierce’s disease resistance in *V. arizonica/candicans* (Agüero et al., 2022), it is tempting to speculate that members of this family of proteins are, at least, involved in the *Xylella* interactions in different host species in addition to olives.

The overall responses of genotypes S215 and S234 have a gene composition that differs from that of S105. The GO analysis of upregulated genes indicates that an intense transcription activity is occurring in plant tissues testing positive by qPCR. Interestingly, the epigenetic control of the plant response is envisaged from the GO analysis of downregulated genes in genotype S105. Indeed, the inhibition of expression of histone H2B or its post-translational modification has been associated with the induction of salicylic acid-mediated response to viruses or fungi (Hu et al., 2014; Zhang et al., 2015). A biotic stress response is supported by a number of transcription factors being mainly upregulated in the S215 and S234 genotypes, among those represented by ethylene-responsive transcription factor ERF071-like (OE9A032259), which was found responsible for tylose formation in *Xylella*-infected grapevines (Zaini et al., 2018; Ingel et al., 2021) and ABR1-like. Conversely, a downregulation of the photosynthesis is observed in both the S215 and S234 genotypes affecting, respectively, the light capture/conversion activity of photosystem II and the dark reactions of the Calvin–Benson cycle likely caused by the diversion of the metabolic processes of these genotypes in the defence direction.

Although, out of the scope of this work, when the RNA-seq datasets were blasted against the plant virus database, sequences of olive leaf yellowing associated virus and olive latent virus 1 were found only in genotype S215, while no viral RNA sequences were detected in S105 and S234 (data not shown). Such finding confirms the lack of seed transmission for the most common viruses infecting olives (Martelli, 2013) and suggests that the wide global distribution of olive viruses is a consequence of the movement of infected cuttings for the vegetative propagation rather than a consequence of the seed dispersion or local vector transmission. Indirectly, this finding confirms that S105 and S234 are seed-originated trees and that S215 is part of a grafted tree whose scion was most like infected, allowing the viruses to translocate and infect the rootstock (genotype S215).

### Artificial inoculations on the selected candidate new resistant genotypes

Artificial inoculations performed for the four selected resistant genotypes confirmed, under controlled conditions and using biological replicates, their interesting resistant features as recorded in the field. Interestingly, the only genotypes in which mild symptoms affecting few replicates were recorded are those derived from Leccino crossed with the two local susceptible cultivars (Cellina di Nardò and Ogliarola salentina). Conversely, those crossed with Cipressino, whose preliminary data show that it is not as susceptible as the two aforementioned cultivars (Saponari, personal communication), remained symptomless. With regard to the bacterial population size, it should be remarked that these are small potted plants in which the whole vegetation was subjected to multiple events of transmission (insects caged for 7 days), which may explain why the population and the distribution of the infections are higher than those generally recorded in the field. In this regard, it is worth noting that genotype S105, which appears to activate different mechanisms of response to *Xfp* infections compared to S215 and S234, upon vector inoculation, showed the highest values of bacterial population among the four genotypes tested. Surprisingly, infection in S215 did not progress with time, likely inhibiting *Xfp* spread.

Conclusively, genetic and transcriptomic analyses indicate that resistance to *Xfp* in olives is controlled through the involvement of different gene pathways, as exemplified by the gene expression profiles recovered in the spontaneous genotypes S234 and S215 compared to those of S105 and that these genetic traits are mainly inherited by the cv. Leccino. The present work further confirms previous evidence and indicates that this cultivar represents a good candidate parental line for breeding programs towards extending the panel of currently available *Xfp*-resistant genotypes. More importantly, our work contributes to the identification of potential target genes for biotechnological approaches based on genome editing techniques as well as the identification of spontaneous seedlings that, following the standard procedure for the registration of new varieties, will extend the list of resistant cultivars currently available. Nevertheless, the availability of these selected resistant genotypes will be exploited to generate new genomic data supporting the implementation of the olive genome resources that are currently very limited, especially for the *Xylella*-resistant olive cultivars, hindering the identification of genetic determinants.

## Materials and methods

### Phenotype assessment

In 2016, prospecting surveys were carried out in the *Xylella*-epidemic area of Apulia, namely, in the Salento Peninsula, to search for olive trees surviving the devastating impact of the *Xfp* infections. This is the area where the first European outbreak of *Xylella* was reported in 2013. Since then, millions of trees of the cultivar Cellina di Nardò and Ogliarola salentina, the predominant cultivars grown in this area, succumbed to the infections, developing the typical OQDS. Inspections were focused on trees or olive shrubs located close to severely affected olive groves but apparently free from the typical desiccation phenomenon associated with *Xfp* infections, i.e., displaying promising phenotypic traits of resistance to bacterial infections. Surveys and sampling were carried out all year-round, with most of the field surveys performed in late summer/autumn when symptoms are more evident and trees can be also assessed for olive production and fruit characteristics.

Surveys were prioritized in the area surrounding the first outbreak (discovered in 2013), where infections had a longer history compared to areas where the infections expanded and progressed during this decade. To this end, locations were empirically categorized into three different classes according to the year when infections were firstly discovered, with “3” corresponding to the first outbreak areas and “1” to the most distant areas from the initial outbreaks where infections have been detected only recently (border of the demarcated infected area) (**Supplementary Figure S1**).

Most of the genotypes were located in non-cultivated areas (spots of “Mediterranean maquis”) on the borders of orchards or roads. Only a few consisted of plants i) grown within olive groves or gardens or ii) re-grown from the rootstocks of ancient trees of Cellina di Nardò or Ogliarola salentina, whose canopy was completely dead. To increase the efficacy of the visual selection of the genotypes, only plants in the adult stage were considered in our surveys. In contrast, plants in the juvenile stage were excluded, both for the short time of exposure to the infections and for the lack of symptom development in young plants. Finally, 171 spontaneous genotypes were retained and monitored for several years. Throughout the field assessments, *Xfp*-induced symptoms were observed, and these were recorded using an empirical scale from 0 to 5 (0 when the tree did not show any desiccation and 5 when the whole canopy was severely affected) (**Table 3**).

**Table 3:** Criteria used to categorize the field-selected genotypes. .

Classification of the tree	Symptom score	Estimation of the bacterial population (average of the results on the subsample)	Distribution of bacterium in the canopy (% of the subsamples testing positive)
**Highly susceptible (HS)**	>3.5	10^5^–10^6^ CFU/mL	>80%
**Susceptible (S)**	2.5–3.5	10^5^–10^6^ CFU/mL	>80%
**Tolerant (T)**	<2	10^4^–10^5^ CFU/mL	>50%
**Resistant (R)**	<2	10^3^–10^4^ CFU/mL	<40%
**Highly resistant (HR)**	<1	10^3^–10^4^ CFU/mL	<25%

### Diagnostic tests for *X. fastidiosa*

From each asymptomatic genotype, a representative sample consisting of several branches and shoots was collected. When the first diagnostic test yielded negative results (i.e., the bacterium was not detectable or present in the samples) or at low concentrations (high quantitation cycles), the test was repeated by testing from four up to eight subsamples/genotype (n. of sampled varied according to the size of the trees) to gather validated data on the presence and distribution of the bacterium on the canopy (**Table 3**). Genotypes testing negative or with low bacterial populations were subjected to further visual inspections and diagnostic tests for the following years to assess the durability of the potential resistant phenotype.

Diagnostic tests were carried out by qPCR assay designed to target a region of the rimM gene (Harper et al., 2010). Briefly, total DNA was purified from 0.5 g of plant tissue prepared from semi-hardwood shoots, using the commercial kit Maxwell^®^ RSC PureFood GMO and Authentication Kit (Promega Corporation, Madison, WI, USA). An aliquot of 1.5 µL (containing approx. 82.5 ng of total DNA) of the purified DNA was then used to set the multiplex qPCR reactions in a final volume of 12.5 µL, containing 1× TaqMan Fast Advance Master Mix (Applied Biosystems, Foster City, CA, USA) and the primers targeting both the bacterial gene and those specific to amplify the plant constitutive cytochrome oxidase (COX) gene as internal control (Weller et al., 2000). Both sets of primers were used at 300 µM of concentration, while the TaqMan probes were at 200 µM (**Supplementary Table S16**). Each run included three replicates of a serial standard dilution containing from 10^7^ to 10 CFU/mL for generating a calibration curve used to estimate the bacterial populations in the *Xylella*-infected samples.

Samples were categorized as positive when the quantitation cycle (Cq) produced was lower than 34 and negative when Cq values were higher or not detected.

### Categorization of the spontaneous trees

Field and laboratory data were used to categorize the spontaneous genotypes into the following classes: HR, R, T, S, and HS (**Supplementary Figures S1A, C**; **Table 3**). Briefly, categorization was based on symptoms, estimation of the bacterial population size, and the frequency of infected shoots/branches on the canopy.

### DNA extraction and SSR genotyping

Genomic DNA was extracted from fresh leaves using a plant DNA purification kit (Exgene Plant SV mini, GeneAll, Seoul, Korea) according to the manufacturer’s instructions. For genetic analysis, 10 highly polymorphic SSR markers were used, including DCA3-5-9-16-18, EMO90, GAPU71B-101-103A, and UDO-043, which were previously selected as the best-performing loci (Baldoni et al., 2009; Haouane et al., 2011) and have also been used in several olive genotyping studies (Mousavi et al., 2017). PCR amplifications were performed in a final volume of 25 µL containing 25 ng DNA, 10× PCR buffer, 200 μM each dNTP, 10 pmol of each forward and reverse primer, and 2U of Q5 High-Fidelity DNA Polymerase (New England Biolabs, Ipswich, MA, USA) (**Supplementary Table S16**). All amplifications were performed under the following conditions: 5 min at 95°C, 35 cycles consisting of 25 sec at 95°C, 30 sec at the appropriate annealing temperature, 25 sec at 72°C, and final elongation at 72°C for 40 min. To distinguish alleles, fluorescent fragments were resolved by capillary electrophoresis in an ABI 3130 Genetic Analyzer (Applied Biosystems-Hitachi) using the internal GeneScan™ 500 LIZ Size Standard (Applied Biosystems^®^, Thermo Fisher Scientific, Waltham, MA, USA). Sample analyses were performed using GeneMapper genotyping software v5 (Applied Biosystems-Hitachi).

SSR data of spontaneous genotypes were compared with those of a wide representative sample of Mediterranean and beyond cultivars previously published (Trujillo et al., 2014; Mousavi et al., 2017; El Bakkali et al., 2019; Valeri et al., 2022) for a total of 653 genotypes.

### Frequency analysis and genetic differentiation

For each SSR locus, the number of alleles (Na), effective alleles (Ne), Shannon’s information index (I), observed (Ho), and expected (He) heterozygosity and fixation index (F) were calculated using GenAlEx 6.5 (Peakall and Smouse, 2012). PIC was calculated for each microsatellite locus using CERVUS version 3.0.3 (Kalinowski et al., 2007; Marshall et al., 1998). To determine the genetic relationships among spontaneous olive accessions with national and international olive varieties, a triangular genetic matrix was performed by GenAlEx 6.5, and the cluster analysis with the NJ method was displayed by the Mega 7 software (Kumar et al., 2016). The final dendrograms were drawn by Figtree 1.4.4 (tree.bio.ed.ac.uk/software/figtree/) to better visualize the placement of each spontaneous olive tree.

Bayesian model-based cluster analysis was also performed for SSR data using STRUCTURE v.2.3.4 software (Pritchard et al., 2000) to identify genetic populations. For cluster values from K = 1 to K = 10, an admixture model and independent allele frequency model (no prior information was used to define clusters) were used to perform a Markov chain Monte Carlo (MCMC) simulation algorithm. The length of the burn-in period was set to 10,000; MCMC after the burn-in period was set to 10,000, and for each K value, the calculation was repeated 20 times. The method of Evanno (Evanno et al., 2005) was used to determine the optimal K value. The program Structure Harvester v.0.9.94 website was used to calculate the optimal value of K using the deltaK criterion (Earl and vonHoldt, 2012).

Paternity was determined for spontaneous olives using the maximum likelihood-based method described in Kalinowski et al. (2007) and implemented in CERVUS version 3.0.3 (Marshall et al., 1998; Kalinowski et al., 2007). Logarithm of the odds (LOD) scores were computed for each sample to assign the best candidate parent with a 95% confidence interval. Ten thousand offspring were simulated, allowing for selfing and using the following parameters: the number of candidate fathers was the number of total analysed varieties in this study. The proportions of “typed loci”, “mistyped loci”, and “minimum typed loci” were always set at 0.95, 0.05, and 9, respectively, with a relaxed value of 85% and a strict level of 95%.

### Plant materials for transcriptomic profiling of selected olive genotypes

Genotypes S105, S215, and S234, categorized as HR and R and showing interesting agronomic features, were selected and sampled to recover the RNA fractions for high-throughput sequencing. S105 was a tree located on the border of a plot with horticultural crops and olives, S234 was grown in an olive grove of Cellina di Nardò, and S215 corresponded to the vigorous and productive vegetation developed below the grafting point of a century-old tree of Ogliarola salentina, whose canopy was completed dead.

Given that each genotype was available as an individual single tree, multiple samples were collected from the same tree, and the recovered xylem tissues were used to extract both the DNA and RNA fractions. Total DNA fractions were used to assess the presence of bacterial DNA using the same qPCR assay described above, while the purified RNA was used for library construction.

### RNA-seq library preparation and sequencing

Based on qPCR results (i.e., presence/absence of detectable bacterial DNA), five samples for each genotype were then processed for RNA-seq analysis, including at least one sample testing positive in qPCR. Total RNAs were extracted from 1.5 g of xylem tissues that were scraped from debarked olive twigs of approx. 0.5 cm in diameter. Tissues were powdered in liquid nitrogen and immediately stored at −80°C until RNA extraction was performed according to Giampetruzzi et al. (2016). RNA concentrations were determined by spectrophotometry using a NanoDrop apparatus (Thermo Fisher Scientific, Waltham, MA, USA). The integrity of the RNA was assessed by 1% agarose gel.

One microgram of total RNA was used for cDNA synthesis using Illumina’s Stranded mRNA Sample Prep Kit followed by library preparation following Illumina’s guidelines for the TruSeq Stranded mRNA LT sample prep kit. Products were then sequenced using an Illumina NovaSeq platform (Illumina, San Diego, CA, USA) on a 100-bp paired-end run.

### RNA-seq data processing and first exploratory data analysis

The FastQC tool was used to assess the read quality of the raw reads. RNA-seq paired-end reads obtained by the sequencing were aligned using the TopHat2 tool (Kim et al., 2013) on the olive genome of *O. europaea* L. subsp. *europaea* var. *europaea* cv. Farga, publicly released into the National Center for Biotechnology Information (NCBI) (Cruz et al., 2016; Julca et al., 2020). Raw read count data from the mapping genome were determined using SeqMonk software version 1.48.1 (https://www.bioinformatics.babraham.ac.uk/projects/seqmonk/), and the resulting abundance data were analysed first using the pcaExplorer R package (Marini and Binder, 2019) in order to cluster the samples according to their genotype (genotype) and Xf_DDpos/Xf_neg status (condition). Genes with less than 1 count summed up across all samples were excluded from further analysis.

PCA was carried out based on the normalized gene expression variance-stabilizing transformation (VST) from the RNA-seq datasets with the samples clustered according to their condition (Xf_DDpos/Xf_neg) and genotype (S105, S215, and S234). Hierarchical clustering data analysis on most variable genes (3,000 genes/transcripts) was performed in R using the pcaExplorer package. Clustering analysis was performed using Euclidean distances and the complete linkage method.

### Differential gene expression analysis

Gene/transcript expression levels in the RNA-seq analysis were measured as raw count reads per transcript by SeqMonk. The DESeq2 package version 1 software was used to identify DEGs on the identified clusters, considering genes annotated in the cv. Farga genome. Differential gene expression analysis was performed via pairwise comparisons between the cluster of libraries of infected samples (Xf_DDpos) and the cluster of libraries on healthy samples (Xf_neg).

For all genotypes, differential gene expression analysis was conducted between the two classes Xf_neg and Xf_DDpos as indicated in **Table 2**. Among all the obtained DEGs, those having a decreased log2 fold change greater than 2 or less than −2 with an adjusted p-value of less than 0.001 and the Benjamini–Hochberg FDR (FDR < 0.01) were considered for further analysis.

Functional enrichment analysis of GO was performed to identify the significant terms and pathways filtered according to the Bonferroni-corrected p-value <0.05. GO enrichment terms and pathways were analysed using the ShinyGO and agriGO v2.0 tools (http://systemsbiology.cpolar.cn/agriGOv2/). In addition, the differential expression profiles were mapped to the metabolic regulatory pathway in detail using MapMan4 (Bolger et al., 2021). Considering that the MapMan software lacks the mapping file from the *O. europaea* reference genome, firstly, the coding sequences were extracted from cv. Farga genome annotations and then uploaded to Mercator4 v.2.0 (https://www.plabipd.de/mercator_main.html) for gene annotation and to obtain the corresponding mapping file. Then, the mapping file containing the gene expression value was imported into MapMan and metabolism overview; maps were drawn for each genotype using the MapMan software.

### Artificial inoculation of four selected genotypes

To confirm the phenotypic responses recorded under field conditions on the individual trees, the same Leccino-bearing genotypes used for transcriptomic analysis, plus genotype S218 (a cross between Leccino and Cellina di Nardò), were propagated and artificially inoculated to monitor the phenotypic response to the infection under controlled conditions. Briefly, 10 replicates for each genotype were obtained by grafting, and when shoots reached 20–30 cm in length, the whole plants were individually caged with 10 P*. spumarius* adults previously maintained for 1 week on *Xfp*-infected olive plants for bacterial acquisition. After 10 days of transmission, the insects were removed, and the plants were maintained in a greenhouse under controlled temperature conditions (25°C–30°C). The panel of plants included the same number of replicates of cultivars Cellina di Nardò (S) and Leccino (R). Plants were firstly tested by collecting four leaves 8 months after the transmission and then re-sampled at 16 and 24 months by collecting small portions of shoots. Diagnostic tests by qPCR were carried out as previously described. Plants were also continuously inspected for the expression of symptoms.

## Data Availability

The data presented in the study are deposited in the NCBI BioProject repository, accession number PRJNA993167.

## References

[B1] Agüero C. B. Riaz S. Tenscher A. C. Bistué C. Walker M. A. (2022). Molecular and functional characterization of two RGA type genes in the PdR1b locus for Pierce’s disease resistance in Vitis arizonica/candicans. Plant Cell Tissue Organ Cult. 151, 497–510. doi: 10.1007/s11240-022-02366-6

[B2] Almagro L. Gómez Ros L. V. Belchi-Navarro S. Bru R. Ros Barceló A. Pedreño M. A. (2009). Class III peroxidases in plant defence reactions. J. Exp. Bot. 60, 377–390. doi: 10.1093/jxb/ern277 19073963

[B3] Arias-Calderón R. León L. Bejarano-Alcázar J. Belaj A. de la Rosa R. Rodríguez-Jurado D. (2015). Resistance to Verticillium wilt in olive progenies from open-pollination. Sci. Hortic. (Amsterdam). 185, 34–42. doi: 10.1016/j.scienta.2015.01.015

[B4] Atrouz K. Bousba R. Marra F. P. Marchese A. Conforti F. L. Perrone B.. (2021). Algerian olive germplasm and its relationships with the central-western mediterranean varieties contributes to clarify cultivated olive diversification. Plants 10, 678. doi: 10.3390/plants10040678 33916098 PMC8066573

[B5] Baldoni L. Cultrera N. G. Mariotti R. Ricciolini C. Arcioni S. Vendramin G. G.. (2009). A consensus list of microsatellite markers for olive genotyping. Mol. Breed. 24, 213–231. doi: 10.1007/s11032-009-9285-8

[B6] Baptista P. Cameirão C. Giampetruzzi A. Morelli M. Abou Kubaa R. Altamura G.. (2019). Understanding the olive microbiome of susceptible and resistant cultivars for sustainable biocontrol. J. Plant Pathol. doi: 10.5281/ZENODO.3552272

[B7] Bazakos C. Manioudaki M. E. Sarropoulou E. Spano T. Kalaitzis P. (2015). 454 pyrosequencing of olive (Olea europaea L.) transcriptome in response to salinity. PloS One 10, e0143000. doi: 10.1371/journal.pone.0143000 26576008 PMC4648586

[B8] Bolger M. Schwacke R. Usadel B. (2021). MapMan visualization of RNA-seq data using mercator4 functional annotations. Methods Mol. Biol. 2354, 195–212. doi: 10.1007/978-1-0716-1609-3_9 34448161

[B9] Boscia D. Altamura G. Notte P. Morelli M. Saldarelli P. Saponari M.. (2017). Resistenza a Xylella fastidiosa in diverse cultivar di olivo. L’ Inf. Agrar. 11. doi: 10.5281/zenodo.495708

[B10] Caarls L. Elberse J. Awwanah M. Ludwig N. R. De Vries M. Zeilmaker T.. (2017). Arabidopsis JASMONATE-INDUCED OXYGENASES down-regulate plant immunity by hydroxylation and inactivation of the hormone jasmonic acid. Proc. Natl. Acad. Sci. 114, 6388–6393. doi: 10.1073/pnas.1701101114 28559313 PMC5474790

[B11] Cabanás C. G. L. Schilirò E. Valverde-Corredor A. Mercado-Blanco J. (2015). Systemic responses in a tolerant olive (Olea europaea L.) cultivar upon root colonization by the vascular pathogen Verticillium dahliae. Front. Microbiol. 6. doi: 10.3389/fmicb.2015.00928 PMC4584997 26441865

[B12] Cariddi C. Saponari M. Boscia D. De Stradis A. Loconsole G. Nigro F.. (2014). Isolation of a Xylella fastidiosa strain infecting olive and oleander in Apulia, Italy. J. Plant Pathol. 96 (3), 1–5. doi: 10.4454/JPP.V96I2.024

[B13] Castillo A. I. Chacón-Díaz C. Rodríguez-Murillo N. Coletta-Filho H. D. Almeida R. P. P. (2020). Impacts of local population history and ecology on the evolution of a globally dispersed pathogen. BMC Genomics 21, 369. doi: 10.1186/s12864-020-06778-6 32434538 PMC7238557

[B14] Catinot J. Huang J. B. Huang P. Y. Tseng M. Y. Chen Y. L. Gu S. Y.. (2015). ETHYLENE RESPONSE FACTOR 96 positively regulates Arabidopsis resistance to necrotrophic pathogens by direct binding to GCC elements of jasmonate - and ethylene-responsive defence genes. Plant Cell Environ. 38, 2721–2734. doi: 10.1111/pce.12583 26038230

[B15] Cheng Z. Zhan M. Yang Z. Zumstein K. Chen H. Huang Q. (2017). The major qualitative characteristics of olive (Olea europaea L.) cultivated in southwest China. Front. Plant Sci. 8. doi: 10.3389/fpls.2017.00559 PMC5437209 28579990

[B16] Choi H.-K. Iandolino A. da Silva F. G. Cook D. R. (2013). Water Deficit Modulates the Response of Vitis vinifera to the Pierce’s Disease Pathogen Xylella fastidiosa. Mol. Plant-Microbe Interact. 26, 643–657. doi: 10.1094/MPMI-09-12-0217-R 23425100

[B17] Coletta-Filho H. Francisco C. S. Lopes J. R. S. De Oliveira A. F. Da Silva L. F. (2016). First report of olive leaf scorch in Brazil, associated with Xylella fastidiosa subsp. pauca. Phytopathol. Mediterr. 55, 130–135. doi: 10.14601/Phytopathol_Mediterr-17259

[B18] Cruz F. Julca I. Gómez-Garrido J. Loska D. Marcet-Houben M. Cano E.. (2016). Genome sequence of the olive tree, Olea europaea. Gigascience 5, 29. doi: 10.1186/s13742-016-0134-5 27346392 PMC4922053

[B19] de Pascali M. Vergine M. Sabella E. Aprile A. Nutricati E. Nicolì F.. (2019). Molecular effects of xylella fastidiosa and drought combined stress in olive trees. Plants 8, 437. doi: 10.3390/plants8110437 31652681 PMC6918294

[B20] de Toledo Thomazella D. P. Seong K. Mackelprang R. Dahlbeck D. Geng Y. Gill U. S.. (2021). Loss of function of a DMR6 ortholog in tomato confers broad-spectrum disease resistance. Proc. Natl. Acad. Sci. U.S.A. 118. doi: 10.1073/pnas.2026152118 PMC8271637 34215692

[B21] Diez C. M. Trujillo I. Martinez-Urdiroz N. Barranco D. Rallo L. Marfil P.. (2015). Olive domestication and diversification in the Mediterranean Basin. New Phytol. 206, 436–447. doi: 10.1111/nph.13181 25420413

[B22] Duran S. T. Aghayeva S. Akparov Z. Mammadov A. Asgarova R. Uslu O. Y.. (2022). Genetic variation and relationships between Azerbaijani and Turkish olive genetic resources. Mol. Biol. Rep. 49, 5209–5217. doi: 10.1007/s11033-021-06564-x 34291396

[B23] Earl D. A. vonHoldt B. M. (2012). STRUCTURE HARVESTER: A website and program for visualizing STRUCTURE output and implementing the Evanno method. Conserv. Genet. Resour. 4, 359–361. doi: 10.1007/s12686-011-9548-7

[B24] El Bakkali A. Essalouh L. Tollon C. Rivallan R. Mournet P. Moukhli A.. (2019). Characterization of worldwide olive germplasm banks of Marrakech (Morocco) and Córdoba (Spain): Towards management and use of olive germplasm in breeding programs. PloS One 14, e0223716. doi: 10.1371/journal.pone.0223716 31622375 PMC6797134

[B25] Evanno G. Regnaut S. Goudet J. (2005). Detecting the number of clusters of individuals using the software STRUCTURE: A simulation study. Mol. Ecol. 14, 2611–2620. doi: 10.1111/j.1365-294X.2005.02553.x 15969739

[B26] Giacomelli L. Zeilmaker T. Scintilla S. Salvagnin U. Voort J.R.v. d. Moser C. (2022). Vitis vinifera plants edited in DMR6 genes show improved resistance to downy mildew. bioRxiv. doi: 10.1101/2022.04.19.488768

[B27] Giampetruzzi A. Baptista P. Morelli M. Cameirão C. Neto T. L. Costa D.. (2020). Differences in the endophytic microbiome of olive cultivars infected by xylella fastidiosa across seasons. Pathogens. 9 (9), 723. doi: 10.3390/pathogens9090723 32887278 PMC7558191

[B28] Giampetruzzi A. Morelli M. Saponari M. Loconsole G. Chiumenti M. Boscia D.. (2016). Transcriptome profiling of two olive cultivars in response to infection by the CoDiRO strain of Xylella fastidiosa subsp. pauca. BMC Genomics 17, 475. doi: 10.1186/s12864-016-2833-9 27350531 PMC4924284

[B29] Grasso F. Coppola M. Carbone F. Baldoni L. Alagna F. Perrotta G.. (2017). The transcriptional response to the olive fruit fly (Bactrocera oleae) reveals extended differences between tolerant and susceptible olive (Olea europaea L.) varieties. PloS One 12, 1–19. doi: 10.1371/journal.pone.0183050 PMC5552259 28797083

[B30] Guerra D. Lamontanara A. Bagnaresi P. Orrù L. Rizza F. Zelasco S.. (2015). Transcriptome changes associated with cold acclimation in leaves of olive tree (Olea europaea L.). Tree Genet. Genomes 11, 113. doi: 10.1007/s11295-015-0939-x

[B31] Haouane H. El Bakkali A. Moukhli A. Tollon C. Santoni S. Oukabli A.. (2011). Genetic structure and core collection of the World Olive Germplasm Bank of Marrakech: Towards the optimised management and use of Mediterranean olive genetic resources. Genetica 139, 1083–1094. doi: 10.1007/s10709-011-9608-7 21960415 PMC3247671

[B32] Harper S. J. Ward L. I. Clover G. R. G. (2010). Development of LAMP and real-time PCR methods for the rapid detection of Xylella fastidiosa for quarantine and field applications. Phytopathology 100, 1282–1288. doi: 10.1094/PHYTO-06-10-0168 20731533

[B33] Hosseini-Mazinani M. Mariotti R. Torkzaban B. Sheikh-Hassani M. Ataei S. Cultrera N. G. M.. (2014). High genetic diversity detected in olives beyond the boundaries of the Mediterranean sea. PloS One 9, e93146. doi: 10.1371/journal.pone.0093146 24709858 PMC3977848

[B34] Hu M. Pei B.-L. Zhang L.-F. Li Y.-Z (2014). Histone H2B monoubiquitination is involved in regulating the dynamics of microtubules during the defense response to Verticillium dahliae toxins in arabidopsis. Plant Physiol. 164, 1857–1865. doi: 10.1104/pp.113.234567 24567190 PMC3982748

[B35] Hussain M. Naz S. Rameez Khan M. Awan A. A. Hussain M. Ali S. (2019). Diversity and divergence in cultivated and wild olive germplasm collected from Northern Pakistan. Int. J. Agric. Biol. 2019, 1109–1115. doi: 10.17957/IJAB/15.1176

[B36] Ingel B. Reyes C. Massonnet M. Boudreau B. Sun Y. Sun Q.. (2021). Xylella fastidiosa causes transcriptional shifts that precede tylose formation and starch depletion in xylem. Mol. Plant Pathol. 22, 175–188. doi: 10.1111/mpp.13016 33216451 PMC7814960

[B37] Islam A. S. M. F. Sanders D. Mishra A. K. Joshi V. (2021). Genetic diversity and population structure analysis of the usda olive germplasm using genotyping-by-sequencing (Gbs). Genes (Basel). 12, 2007. doi: 10.3390/genes12122007 34946959 PMC8701156

[B38] Jiménez-Ruiz J. Ramírez-Tejero J. A. Fernández-Pozo N. de la O Leyva-Pérez M. Yam H. de la Rosa R.. (2020). Transposon activation is a major driver in the genome evolution of cultivated olive trees (Olea europaea L.). Plant Genome 13. doi: 10.1002/tpg2.20010 PMC12806974 33016633

[B39] Julca I. Marcet-Houben M. Cruz F. Gómez-Garrido J. Gaut B. S. Díez C. M.. (2020). Genomic evidence for recurrent genetic admixture during the domestication of Mediterranean olive trees (Olea europaea L.). BMC Biol. 18. doi: 10.1186/s12915-020-00881-6 PMC7586694 33100219

[B40] Kahn A. K. Almeida R. P. P. (2022). Phylogenetics of historical host switches in a bacterial plant pathogen. Appl. Environ. Microbiol. 88. doi: 10.1128/aem.02356-21 PMC9004383 35311514

[B41] Kalinowski S. T. Taper M. L. Marshall T. C. (2007). Revising how the computer program CERVUS accommodates genotyping error increases success in paternity assignment. Mol. Ecol. 16, 1099–1106. doi: 10.1111/j.1365-294X.2007.03089.x 17305863

[B42] Karamatlou I. Navabpour S. Nezhad K. Z. Mariotti R. Mousavi S. Hosseini-Mazinani M. (2023). Cold stress resilience of Iranian olive genetic resources: evidence from autochthonous genotypes diversity. Front. Plant Sci. 14. doi: 10.3389/fpls.2023.1140270 PMC10204771 37229112

[B43] Khadari B. El Bakkali A. Essalouh L. Tollon C. Pinatel C. Besnard G. (2019). Cultivated olive diversification at local and regional scales: evidence from the genetic characterization of french genetic resources. Front. Plant Sci. 10. doi: 10.3389/fpls.2019.01593 PMC6937215 31921243

[B44] Kieu N. P. Lenman M. Wang E. S. Petersen B. L. Andreasson E. (2021). Mutations introduced in susceptibility genes through CRISPR/Cas9 genome editing confer increased late blight resistance in potatoes. Sci. Rep. 11. doi: 10.1038/s41598-021-83972-w PMC7904907 33627728

[B45] Kim D. Pertea G. Trapnell C. Pimentel H. Kelley R. Salzberg S. L. (2013). TopHat2: Accurate alignment of transcriptomes in the presence of insertions, deletions and gene fusions. Genome Biol. 14. doi: 10.1186/gb-2013-14-4-r36 PMC4053844 23618408

[B46] Koseoglou E. van der Wolf J. M. Visser R. G. F. Bai Y. (2022). Susceptibility reversed: modified plant susceptibility genes for resistance to bacteria. Trends Plant Sci. 27, 69–79. doi: 10.1016/j.tplants.2021.07.018 34400073

[B47] Krivanek A. F. Riaz S. Walker M. A. (2006). Identification and molecular mapping of PdR1 a primary resistance gene to Pierce’s disease in Vitis. Theor. Appl. Genet. 112, 1125–1131. doi: 10.1007/s00122-006-0214-5 16435126

[B48] Krugner R. Sisterson M. S. Chen J. Stenger D. C. Johnson M. W. (2014). Evaluation of olive as a host of Xylella fastidiosa and associated sharpshooter vectors. Plant Dis. 98, 1186–1193. doi: 10.1094/PDIS-01-14-0014-RE 30699616

[B49] Kumar S. Stecher G. Tamura K. (2016). MEGA7: molecular evolutionary genetics analysis version 7.0 for bigger datasets. Mol. Biol. Evol. 33, 1870–1874. doi: 10.1093/MOLBEV/MSW054 27004904 PMC8210823

[B50] Landa B. B. Castillo A. I. Giampetruzzi A. Kahn A. Román-Écija M. Velasco-Amo M. P.. (2020). Emergence of a plant pathogen in europe associated with multiple intercontinental introductions. Appl. Environ. Microbiol. 86 (3). doi: 10.1128/AEM.01521-19 PMC6974645 31704683

[B51] Laranjeira F. F. Pompeu J. Harakava R. Figueiredo J. O. Carvalho S. A. Coletta H. D. (1998). Cultivares e espécies cítricas hospedeiras de Xylella fastidiosa em condição de campo. Fitopatol. Bras. 23, 147–154.

[B52] Leyva-Pérez M. de la O. Jiménez-Ruiz J. Gómez-Lama Cabanás C. Valverde-Corredor A. Barroso J. B.. (2018). Tolerance of olive (Olea europaea) cv Frantoio to Verticillium dahliae relies on both basal and pathogen-induced differential transcriptomic responses. New Phytol. 217, 671–686. doi: 10.1111/nph.14833 29023762

[B53] Liu R. Xu Y. H. Jiang S. C. Lu K. Lu Y. F. Feng X. J.. (2013). Light-harvesting chlorophyll a/b-binding proteins, positively involved in abscisic acid signalling, require a transcription repressor, WRKY40, to balance their function. J. Exp. Bot. 64, 5443–5456. doi: 10.1093/jxb/ert307 24078667 PMC3871805

[B54] Marini F. Binder H. (2019). PcaExplorer: An R/Bioconductor package for interacting with RNA-seq principal components. BMC Bioinf. 20. doi: 10.1186/s12859-019-2879-1 PMC6567655 31195976

[B55] Mariotti R. Belaj A. de la Rosa R. Muleo R. Cirilli M. Forgione I.. (2023). Genealogical tracing of Olea europaea species and pedigree relationships of var. europaea using chloroplast and nuclear markers. BMC Plant Biol. 23. doi: 10.1186/s12870-023-04440-3 PMC10521521 37749509

[B56] Marshall T. C. Slate J. Kruuk L. E. B. Pemberton J. M. (1998). Statistical confidence for likelihood-based paternity inference in natural populations. Mol. Ecol. 7, 639–655. doi: 10.1046/j.1365-294x.1998.00374.x 9633105

[B57] Martelli G. (2013). A brief outline of infectious diseases of olive. Palest. Tech. Univ. Res. J. 1. doi: 10.53671/pturj.v1i1.6

[B58] Mauricio F. N. Soratto T. A. T. Diogo J. A. Boscariol-Camargo R. L. De Souza A. A. Coletta-Filho H. D.. (2019). Analysis of defense-related gene expression in citrus hybrids infected by xylella fastidiosa. Phytopathology 109, 301–306. doi: 10.1094/PHYTO-09-18-0366-FI 30480473

[B59] Montilon V. De Stradis A. Saponari M. Abou Kubaa R. Giampetruzzi A. D’Attoma G.. (2023). Xylella fastidiosa subsp. pauca ST53 exploits pit membranes of susceptible olive cultivars to spread systemically in the xylem. Plant Pathol. 72, 144–153. doi: 10.1111/ppa.13646

[B60] Moralejo E. Gomila M. Montesinos M. Borràs D. Pascual A. Nieto A.. (2020). Phylogenetic inference enables reconstruction of a long-overlooked outbreak of almond leaf scorch disease (Xylella fastidiosa) in Europe. Commun. Biol. 3. doi: 10.1038/s42003-020-01284-7 PMC7547738 33037293

[B61] Morelli M. García-Madero J. M. Jos Á. Saldarelli P. Dongiovanni C. Kovacova M.. (2021). Xylella fastidiosa in olive: A review of control attempts and current management. Microorganisms 9. doi: 10.3390/microorganisms9081771 PMC8397937 34442850

[B62] Mousavi S. Mariotti R. Regni L. Nasini L. Bufacchi M. Pandolfi S.. (2017). The first molecular identification of an olive collection applying standard simple sequence repeats and novel expressed sequence tag markers. Front. Plant Sci. 8. doi: 10.3389/fpls.2017.01283 PMC5515915 28769972

[B63] Mousavi S. Mariotti R. Valeri M. C. Regni L. Lilli E. Albertini E.. (2022). Characterization of differentially expressed genes under salt stress in olive. Int. J. Mol. Sci. 23. doi: 10.3390/ijms23010154 PMC8745295 35008580

[B64] Mousavi S. Stanzione V. Mencuccini M. Baldoni L. Bufacchi M. Mariotti R. (2019). Biochemical and molecular profiling of unknown olive genotypes from central Italy: determination of major and minor components. Eur. Food Res. Technol. 245, 83–94. doi: 10.1007/s00217-018-3142-0

[B65] Niza B. Coletta-Filho H. D. Merfa M. V. Takita M. A. de Souza A. A. (2015). Differential colonization patterns of Xylella fastidiosa infecting citrus genotypes. Plant Pathol. 64, 1259–1269. doi: 10.1111/ppa.12381

[B66] Omri A. Abdelhamid S. Ayadi M. Araouki A. Gharsallaoui M. Gouiaa M.. (2021). The investigation of minor and rare Tunisian olive cultivars to enrich and diversify the olive genetic resources of the country. J. Food Compos. Anal. 95. doi: 10.1016/j.jfca.2020.103657

[B67] Peakall R. Smouse P. E. (2012). GenALEx 6.5: Genetic analysis in Excel. Population genetic software for teaching and research-an update. Bioinformatics 28, 2537–2539. doi: 10.1093/bioinformatics/bts460 22820204 PMC3463245

[B68] Pirrello C. Malacarne G. Moretto M. Lenzi L. Perazzolli M. Zeilmaker T.. (2022). Grapevine DMR6-1 is a candidate gene for susceptibility to downy mildew. Biomolecules 12. doi: 10.3390/biom12020182 PMC8961545 35204683

[B69] Pritchard J. K. Stephens M. Donnelly P. (2000). Inference of population structure using multilocus genotype data. Genetics 155, 945–959. doi: 10.1093/genetics/155.2.945 10835412 PMC1461096

[B70] Riaz S. Tenscher A. C. Rubin J. Graziani R. Pao S. S. Walker M. A. (2008). Fine-scale genetic mapping of two Pierce’s disease resistance loci and a major segregation distortion region on chromosome 14 of grape. Theor. Appl. Genet. 117, 671–681. doi: 10.1007/s00122-008-0802-7 18516585

[B71] Rodrigues C. M. de Souza A. A. Takita M. A. Kishi L. T. MaChado M. A. (2013). RNA-Seq analysis of Citrus reticulata in the early stages of Xylella fastidiosa infection reveals auxin-related genes as a defense response. BMC Genomics 14. doi: 10.1186/1471-2164-14-676 PMC3852278 24090429

[B72] Sakar E. Unver H. Bakir M. Ulas M. Sakar Z. M. (2016). Genetic relationships among olive (Olea europaea L.) cultivars native to Turkey. Biochem. Genet. 54, 348–359. doi: 10.1007/s10528-016-9723-3 26902471

[B73] Salazar-García D. C. Malheiro R. Pereira J. A. Lopéz-Cortés I. (2019). Unexplored olive cultivars from the Valencian Community (Spain): some chemical characteristics as a valorization strategy. Eur. Food Res. Technol. 245, 325–334. doi: 10.1007/s00217-018-3164-7

[B74] Saponari M. Giampetruzzi A. Loconsole G. Boscia D. Saldarelli P. (2019). Xylella fastidiosa in olive in apulia: Where we stand. Phytopathology 109, 175–186. doi: 10.1094/PHYTO-08-18-0319-FI 30376439

[B75] Schneider K. van der Werf W. Cendoya M. Mourits M. Navas-Cortés J. A. Vicent A.. (2020). Impact of Xylella fastidiosa subspecies pauca in European olives. Proc. Natl. Acad. Sci. U.S.A. 117 (17), 9250–9259. doi: 10.1073/pnas.1912206117 32284411 PMC7196823

[B76] Shiu S. H. Bleecker A. B. (2001). Receptor-like kinases from Arabidopsis form a monophyletic gene family related to animal receptor kinases. Proc. Natl. Acad. Sci. U.S.A. 98, 10763–10768. doi: 10.1073/pnas.181141598 11526204 PMC58549

[B77] Sion S. Taranto F. Montemurro C. Mangini G. Camposeo S. Falco V.. (2019). Genetic characterization of apulian olive germplasm as potential source in new breeding programs. Plants 8. doi: 10.3390/plants8080268 PMC6724140 31387331

[B78] Sorkheh K. Khaleghi E. (2016). Molecular characterization of genetic variability and structure of olive (Olea europaea L.) germplasm collection analyzed by agromorphological traits and microsatellite markers. Turkish J. Agric. For. 40, 583–596. doi: 10.3906/tar-1602-27

[B79] Surano A. Abou Kubaa R. Nigro F. Altamura G. Losciale P. Saponari M.. (2022). Susceptible and resistant olive cultivars show differential physiological response to Xylella fastidiosa infections. Front. Plant Sci. 13. doi: 10.3389/fpls.2022.968934 PMC9530328 36204082

[B80] Tolocka P. A. Mattio M. F. Paccioretti M. A. Otero M. L. Roca M. E. Guzmán F. A.. (2017). Xylella Fastidiosa subsp. Pauca ST69 in olive in Argentina. J. Plant Pathol. 99, 803. doi: 10.4454/jpp.v99i3.3965

[B81] Topi D. Guclu G. Kelebek H. Selli S. (2022). Olive oil production in Albania, chemical characterization, and authenticity. Olive Oil - New Perspect. Applications. doi: 10.5772/intechopen.96861

[B82] Tripathi J. N. Ntui V. O. Shah T. Tripathi L. (2021). CRISPR/Cas9-mediated editing of DMR6 orthologue in banana (Musa spp.) confers enhanced resistance to bacterial disease. Plant Biotechnol. J. 19, 1291–1293. doi: 10.1111/pbi.13614 33934462 PMC8313124

[B83] Trujillo I. Ojeda M. A. Urdiroz N. M. Potter D. Barranco D. Rallo L.. (2014). Identification of the Worldwide Olive Germplasm Bank of Córdoba (Spain) using SSR and morphological markers. Tree Genet. Genomes 10, 141–155. doi: 10.1007/s11295-013-0671-3

[B84] Unver T. Wu Z. Sterck L. Turktas M. Lohaus R. Li Z.. (2017). Genome of wild olive and the evolution of oil biosynthesis. Proc. Natl. Acad. Sci. U.S.A. 114. doi: 10.1073/pnas.1708621114 PMC5676908 29078332

[B85] Valeri M. C. Mifsud D. Sammut C. Pandolfi S. Lilli E. Bufacchi M.. (2022). Exploring olive genetic diversity in the maltese islands. Sustainability 14, 10684. doi: 10.3390/su141710684

[B86] Veloso M. M. Simões-Costa M. C. Carneiro L. C. Guimarães J. B. Mateus C. Fevereiro P.. (2018). Olive Tree (Olea europaea L.) diversity in traditional small farms of Ficalho, Portugal. Diversity 10. doi: 10.3390/d10010005

[B87] Vergine M. Meyer J. B. Cardinale M. Sabella E. Hartmann M. Cherubini P.. (2020). The xylella fastidiosa-resistant olive cultivar “leccino“ has stable endophytic microbiota during the olive quick decline syndrome (OQDS). Pathogens. 9 (1), 35. doi: 10.3390/pathogens9010035 PMC7168594 31906093

[B88] Walker N. C. White S. M. McKay Fletcher D. Ruiz S. A. Rankin K. E. De Stradis A.. (2023). The impact of xylem geometry on olive cultivar resistance to Xylella fastidiosa: An image-based study. Plant Pathol. 72, 521–535. doi: 10.1111/ppa.13674

[B89] Wei X. Wang Y. Zhang S. Gu T. Steinmetz G. Yu H.. (2022). Structural analysis of receptor-like kinase SOBIR1 reveals mechanisms that regulate its phosphorylation-dependent activation. Plant Commun. 3, 100301. doi: 10.1016/j.xplc.2022.100301 35529948 PMC9073325

[B90] Weller S. A. Elphinstone J. G. Smith N. C. Boonham N. Stead D. E. (2000). Detection of Ralstonia solanacearum strains with a quantitative, multiplex, real-time, fluorogenic PCR (TaqMan) assay. Appl. Environ. Microbiol. 66, 2853–2858. doi: 10.1128/AEM.66.7.2853-2858.2000 10877778 PMC92083

[B91] Yelli F. Kato T. Nishiuchi T. (2018). The possible roles of AtERF71 in the defense response against the Fusarium graminearum. Plant Biotechnol. 35, 187–192. doi: 10.5511/plantbiotechnology.18.0501b PMC6879373 31819723

[B92] Zaini P. A. Nascimento R. Gouran H. Cantu D. Chakraborty S. Phu M.. (2018). Molecular profiling of pierce’s disease outlines the response circuitry of vitis vinifera to xylella fastidiosa infection. Front. Plant Sci. 9. doi: 10.3389/fpls.2018.00771 PMC6002507 29937771

[B93] Zeilmaker T. Ludwig N. R. Elberse J. Seidl M. F. Berke L. Van Doorn A.. (2015). DOWNY MILDEW RESISTANT 6 and DMR6-LIKE OXYGENASE 1 are partially redundant but distinct suppressors of immunity in Arabidopsis. Plant J. 81, 210–222. doi: 10.1111/tpj.12719 25376907

[B94] Zhang Y. Li D. Zhang H. Hong Y. Huang L. Liu S.. (2015). Tomato histone H2B monoubiquitination enzymes SlHUB1 and SlHUB2 contribute to disease resistance against Botrytis cinerea through modulating the balance between SA- and JA/ET-mediated signaling pathways. BMC Plant Biol. 15, 252. doi: 10.1186/s12870-015-0614-2 26490733 PMC4618151

