# Supplementary material for: A survey in natural olive resources exposed to high inoculum pressure indicates the presence of traits of resistance to Xylella fastidiosa in Leccino offspring
Source: Front Plant Sci. 2024 Sep 30;15:1457831. doi: 10.3389/fpls.2024.1457831 (PMC11471571; doi:10.3389/fpls.2024.1457831)
Supplement: Supplementary file 1 [file DataSheet1.docx]

Supplementary Material

**Supplementary Figures and Tables**

**Supplementary Figure 1.** **Geographic location of 139 olive spontaneous genotype**. **(A)** Localization of the 139 spontaneous selected genotypes during the survey surrounding the first outbreak in Italy (discovered in 2013); genotypes were represented on the map with different colors according to their phenotype: Highly Resistant (HR); Resistant (R); Tolerant (T); Susceptible (S) and Highly Susceptible (HS); **(B)** Number of genotypes were empirically categorized in three different classes according to the year when infections were firstly discovered and to the location: location score “3” assigned to the first outbreak areas (purple), location score “2” to the second area (red) and location score “1” to the most distant areas from the initial outbreaks (orange). **(C)** Distribution of the 139 spontaneous genotypes according to the location’s score and to the phenotype.

**Supplementary Table 1.** Categorization of the selected trees belonging to known cultivars.

**Supplementary Table 2** Indices of genetic diversity of 171 genotypes for each SSR locus

**Supplementary Figure 2.** NJ analysis together with a set of 482 SSR data from cultivars representative of the worldwide olive diversity

**Supplementary Table 3.** Paternity analysis results

**Supplementary Figure 3.** Sample to sample distance analysis by correlation distance method on the RNASeq data mapped on the Farga genome

**Supplementary Figure 4. Visualization of photosynthesis.** MapMan was used to visualize differentially expressed genes (DEGs) involved in photosynthesis process in the three genotypes infected by Xf.

**Supplementary Figure 5. Custom overview of “External stimuli response” BIN by MapMan4 representing genes responding to Abiotic/Biotic stresses.** The different colors represent the shrunked log2 fold change values of the gene expression levels in response to Xf infection, blue and red represent downregulated and upregulated DEGs.

**Supplementary Figure 6. Visualization of differentially expressed “receptor-like kinase” genes with MapMan4**. The receptor kinase BINs 18.4.1 was illustrated according Shiu et al 2001. (A) S105 (B) S215 and (C) S234. The figure was constructed using shrunked log2 fold change of infected versus healthy. Blue represents downregulated DEGs, and red represents upregulated DEGs

**Supplementary Figure 7. MapMan custom overview of the transcription factors (TFs).** TF genes that were differentially expressed under Xf’s infection in the three different genotypes.

**Supplementary Table 4. List of upregulated DEGs (Xf_DDpos vs Xf_neg) in S105 genotype**

**Supplementary Table 5. GO enrichment analysis of upregulated DEGs in S105 genotype**

**Supplementary Table 6. List of upregulated DEGs (Xf_DDpos vs Xf_neg) in S215 genotype**

**Supplementary Table 7. GO enrichment analysis of upregulated DEGs in S215 genotype**

**Supplementary Table 8. List of upregulated DEGs (Xf_DDpos vs Xf_neg) in S234 genotype**

**Supplementary Table 9. GO enrichment analysis of upregulated DEGs in S234 genotype**

**Supplementary Table 10. List of downregulated DEGs (Xf_DDpos vs Xf_neg) in S105 genotype**

**Supplementary Table 11. GO enrichment analysis of DEGs in S105 genotype**

**Supplementary Table 12. List of downregulated DEGs (Xf_DDpos vs Xf_neg) in S215 genotype**

**Supplementary Table 13. GO enrichment analysis of DEGs in S215 genotype**

**Supplementary Table 14. List of downregulated DEGs (Xf_DDpos vs Xf_neg) in S234 genotype**

**Supplementary Table 15. GO enrichment analysis of DEGs in S234 genotype**

**Supplementary Figure 8. Onset of symptoms recorded on few *Xfp*-inoculated replicates 16 months after vector inoculation. (**A) Plant of Cellina di Nardò showing dieback and withering; (B) Plant of Cellina di Nardò with severe desiccation; (C) Plant of the genotype S218 with initial shoot dieback; d) Plants of the genotype S234 showing only defoliation.

**Supplementary Figure 9. Inoculated plants of the putative resistant genotypes at 24 months post vector inoculation.** A) Plants of Leccino (inoculated as control), genotype S215 (C), genotype S234 (F), genotype S218 (D) and genotype S105 (E) with limited symptoms of defoliation. (B) Plant of Cellina di Nardò (inoculated as control) showing desiccations on the entire plants

**Supplementary Table 16.** **List of all the primers used in the study**
