# Supplementary material for: A survey in natural olive resources exposed to high inoculum pressure indicates the presence of traits of resistance to Xylella fastidiosa in Leccino offspring
Source: Front Plant Sci. 2024 Sep 30;15:1457831. doi: 10.3389/fpls.2024.1457831 (PMC11471571; doi:10.3389/fpls.2024.1457831)
Supplement: Supplementary file 2 [file Presentation1.pptx]

## Slide 1
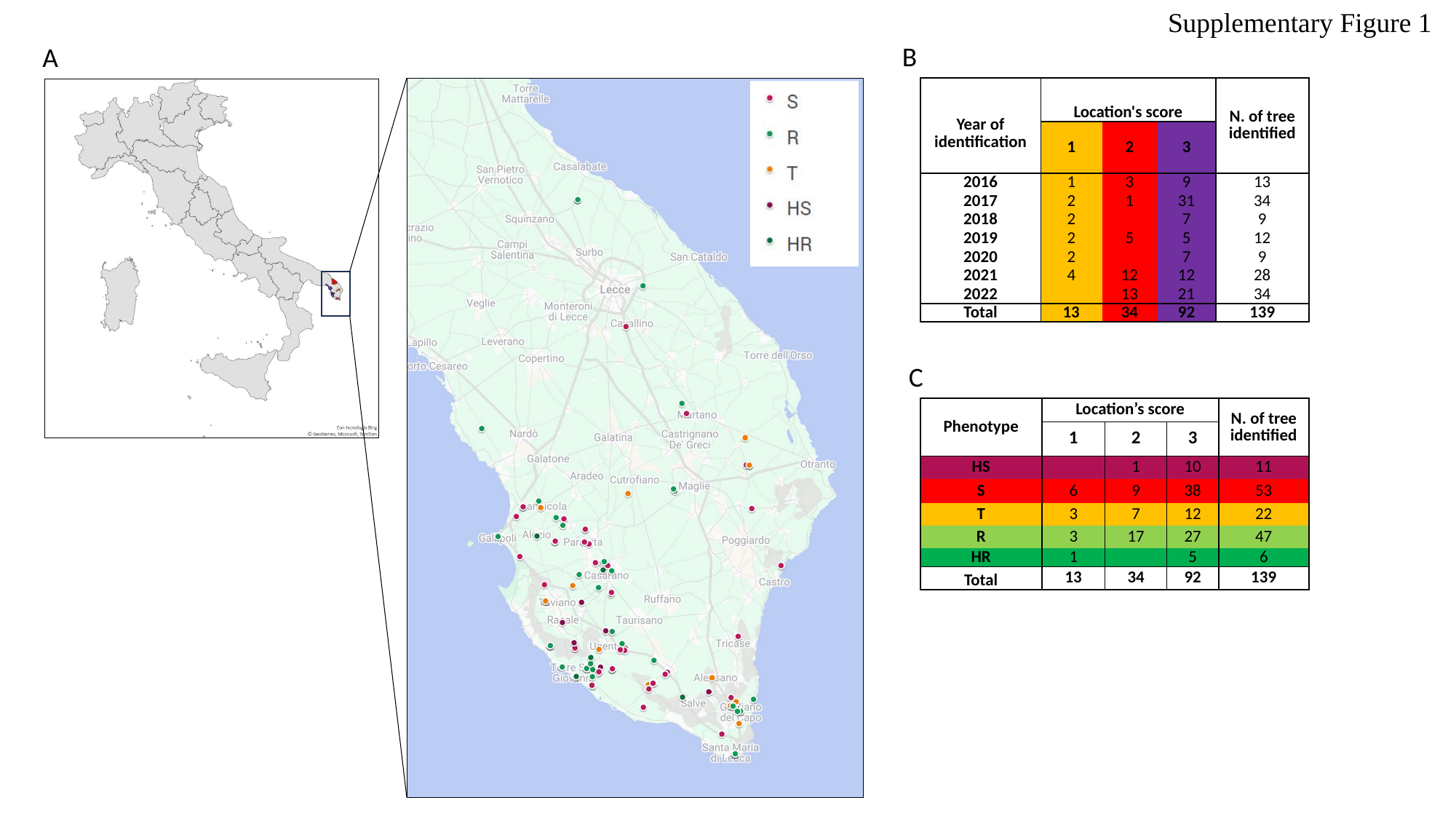

Supplementary Figure 1
B
A
| Year of identification | Location's score | | | N. of tree identified |
| --- | --- | --- | --- | --- |
| Year of identification | 1 | 2 | 3 | |
| 2016 | 1 | 3 | 9 | 13 |
| 2017 | 2 | 1 | 31 | 34 |
| 2018 | 2 | | 7 | 9 |
| 2019 | 2 | 5 | 5 | 12 |
| 2020 | 2 | | 7 | 9 |
| 2021 | 4 | 12 | 12 | 28 |
| 2022 | | 13 | 21 | 34 |
| Total | 13 | 34 | 92 | 139 |
C
| Phenotype | Location’s score | | | N. of tree identified |
| --- | --- | --- | --- | --- |
| | 1 | 2 | 3 | |
| HS | | 1 | 10 | 11 |
| S | 6 | 9 | 38 | 53 |
| T | 3 | 7 | 12 | 22 |
| R | 3 | 17 | 27 | 47 |
| HR | 1 | | 5 | 6 |
| Total | 13 | 34 | 92 | 139 |
