# Supplementary figures and images for: A survey in natural olive resources exposed to high inoculum pressure indicates the presence of traits of resistance to Xylella fastidiosa in Leccino offspring
Source: Front Plant Sci. 2024 Sep 30;15:1457831. doi: 10.3389/fpls.2024.1457831 (PMC11471571; doi:10.3389/fpls.2024.1457831)

## Slide 1
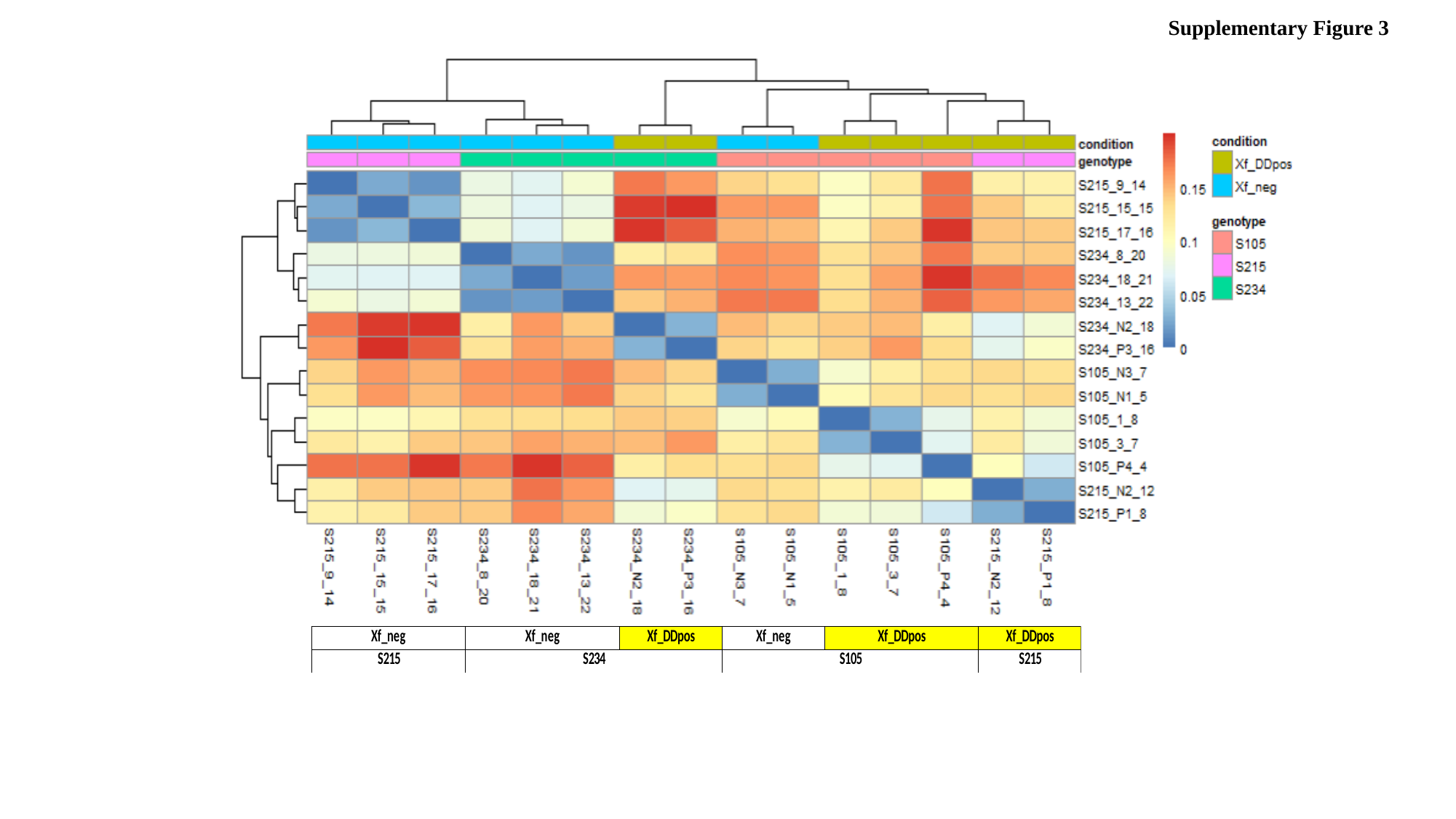

Supplementary Figure 3

Supplement: Supplementary file 4 [file Presentation3.pptx]

## Slide 1
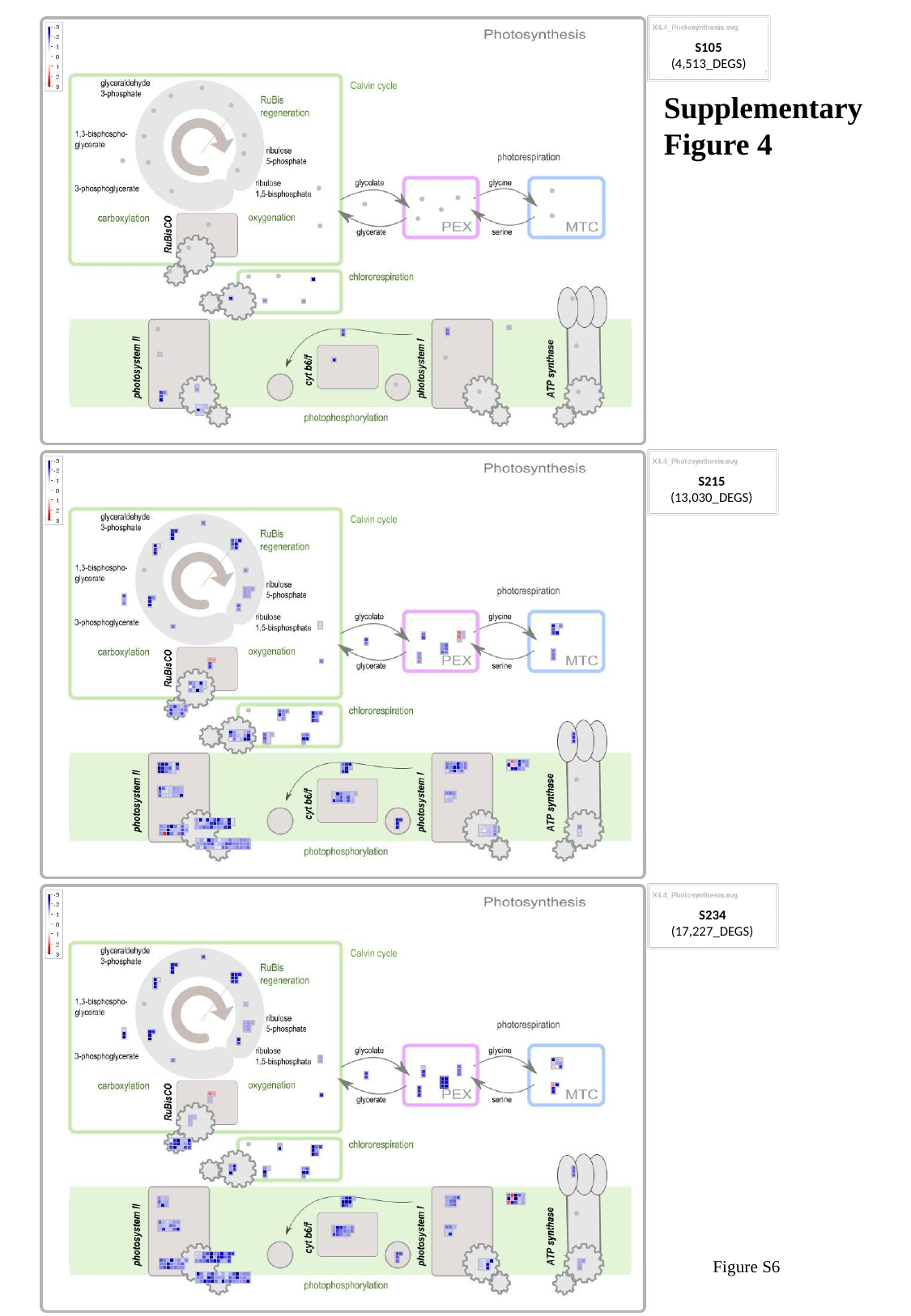

S105
(4,513_DEGS)
S215
(13,030_DEGS)
S234
(17,227_DEGS)
Supplementary
Figure 4
Figure S6

Supplement: Supplementary file 5 [file Presentation4.pptx]

## Slide 1
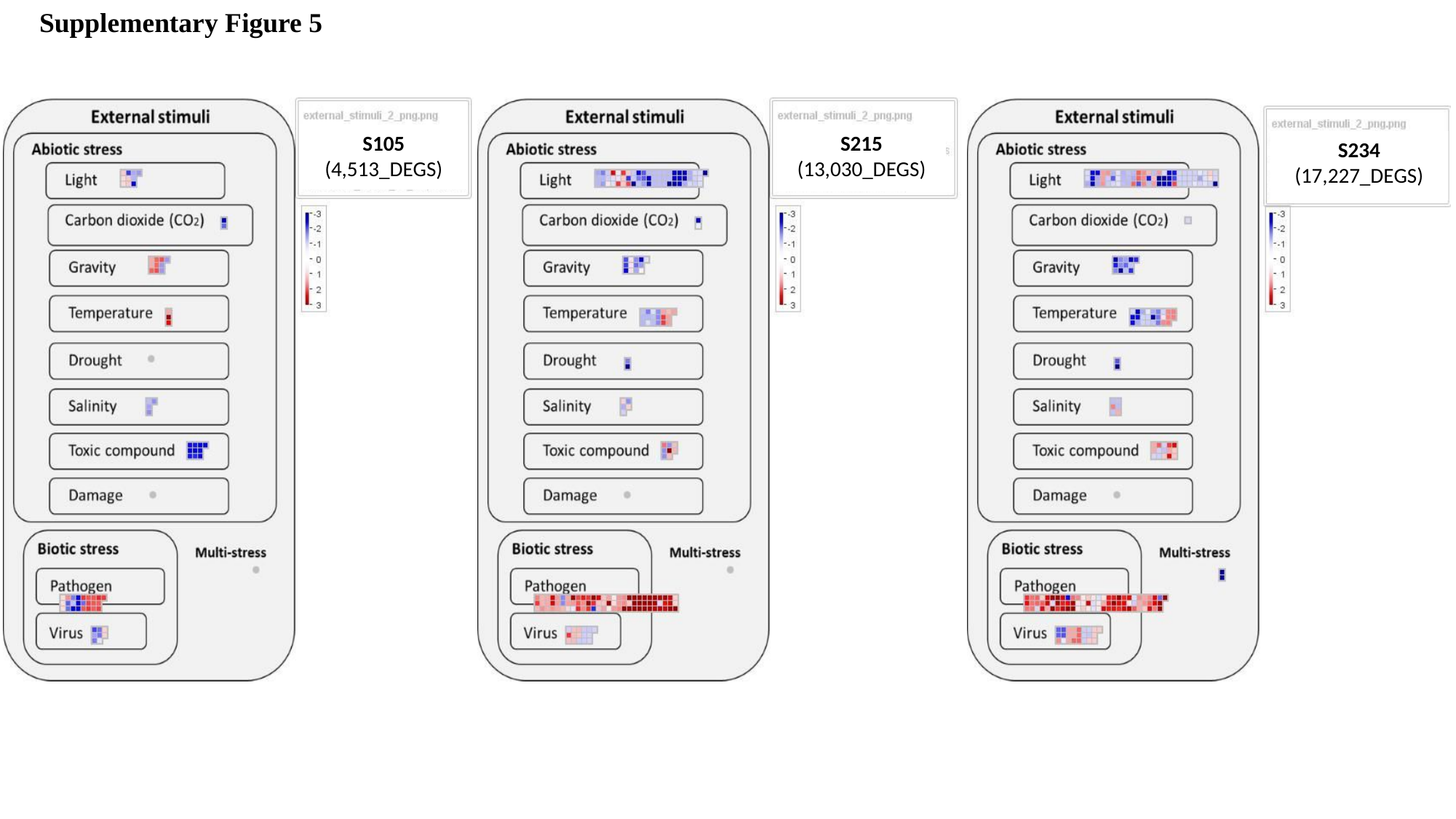

Supplementary Figure 5
S105
(4,513_DEGS)
S215
(13,030_DEGS)
S234
(17,227_DEGS)

Supplement: Supplementary file 6 [file Presentation5.pptx]

## Slide 1
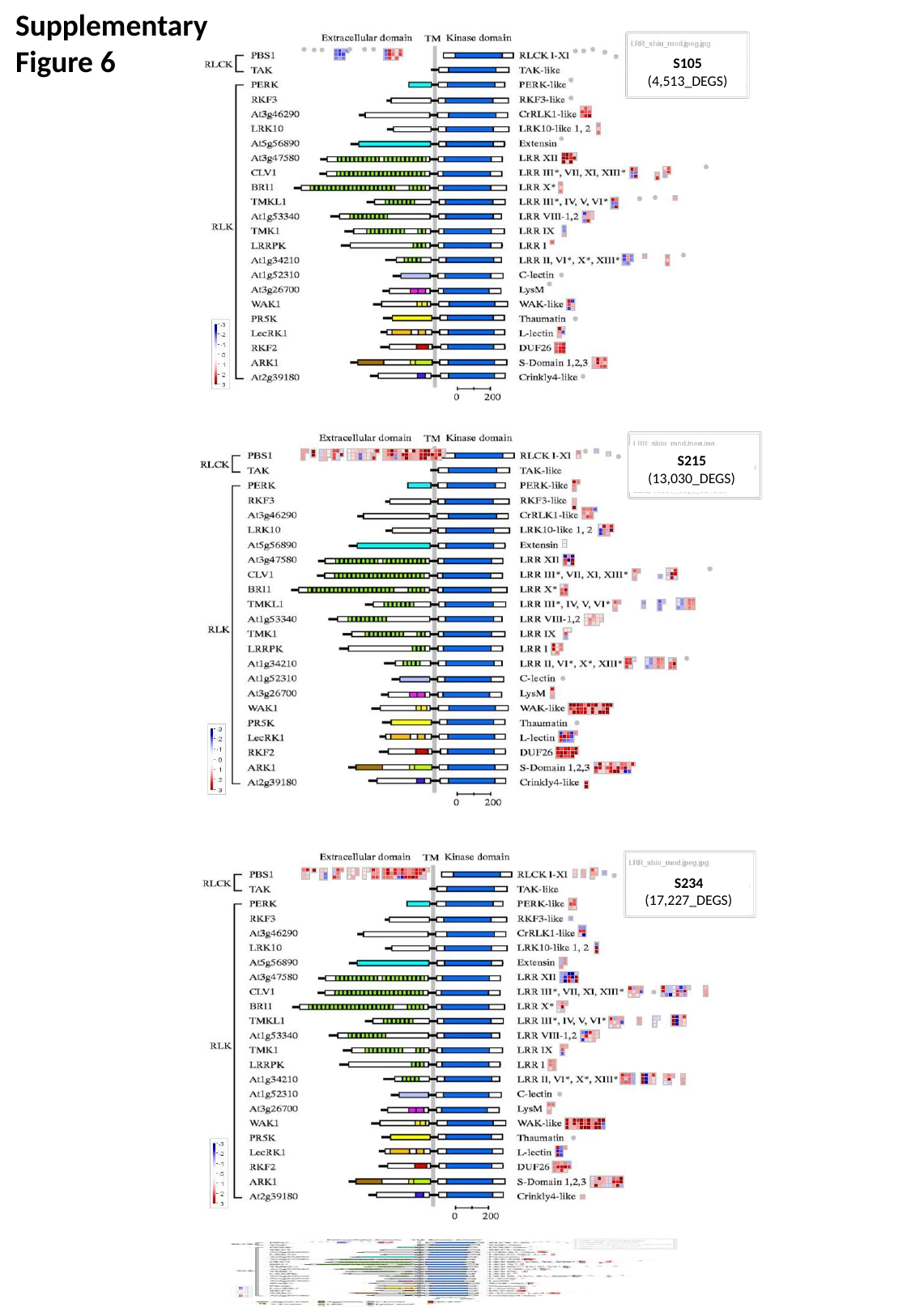

Supplementary
Figure 6
S105
(4,513_DEGS)
S215
(13,030_DEGS)
S234
(17,227_DEGS)

Supplement: Supplementary file 7 [file Presentation6.pptx]

## Slide 1
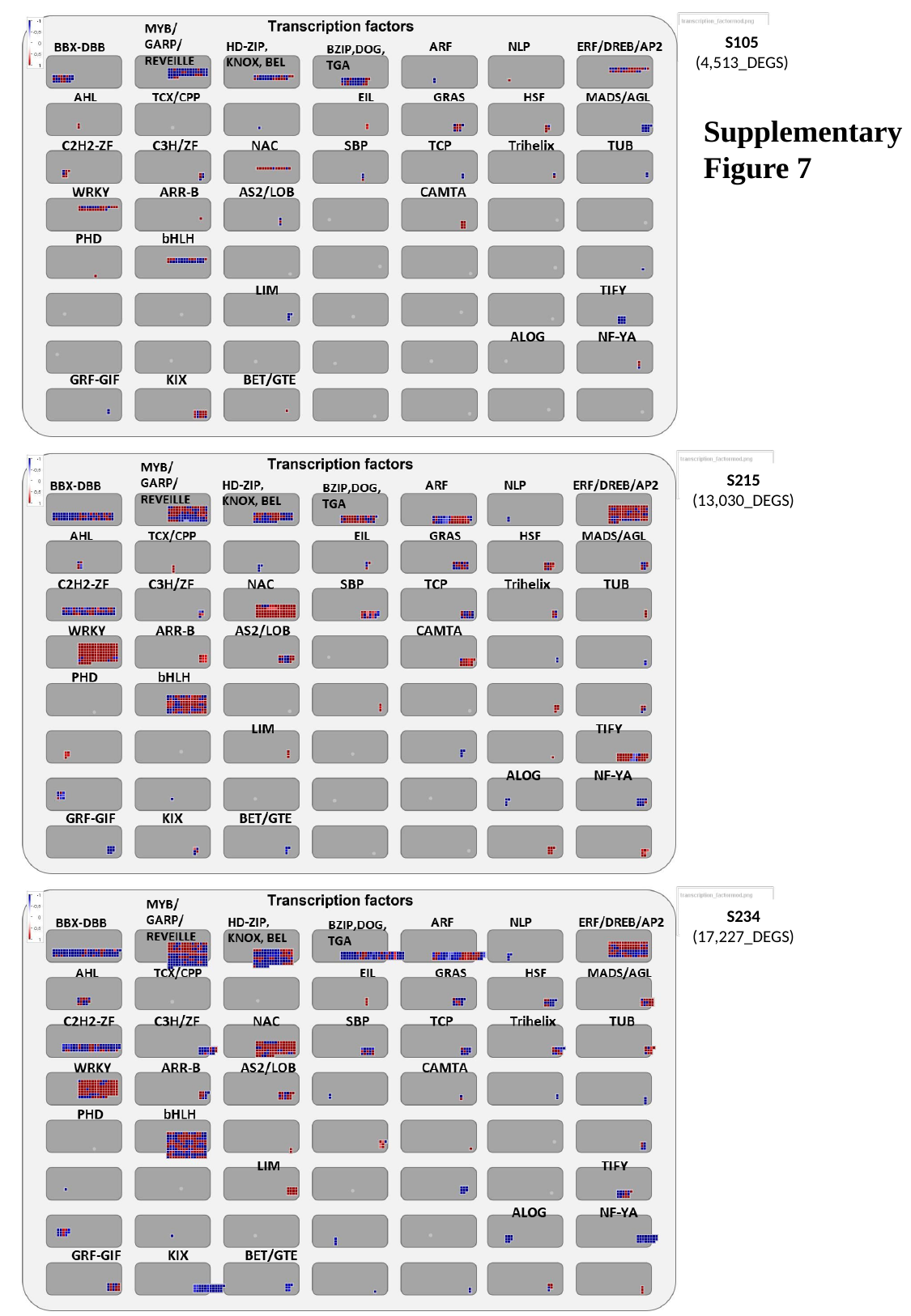

S105
(4,513_DEGS)
Supplementary Figure 7
S215
(13,030_DEGS)
S234
(17,227_DEGS)

Supplement: Supplementary file 8 [file Presentation7.pptx]

**Supplementary Figure 9.** Symptoms recorded on the Xfp-infected plants 24 months (24mvp*i*)

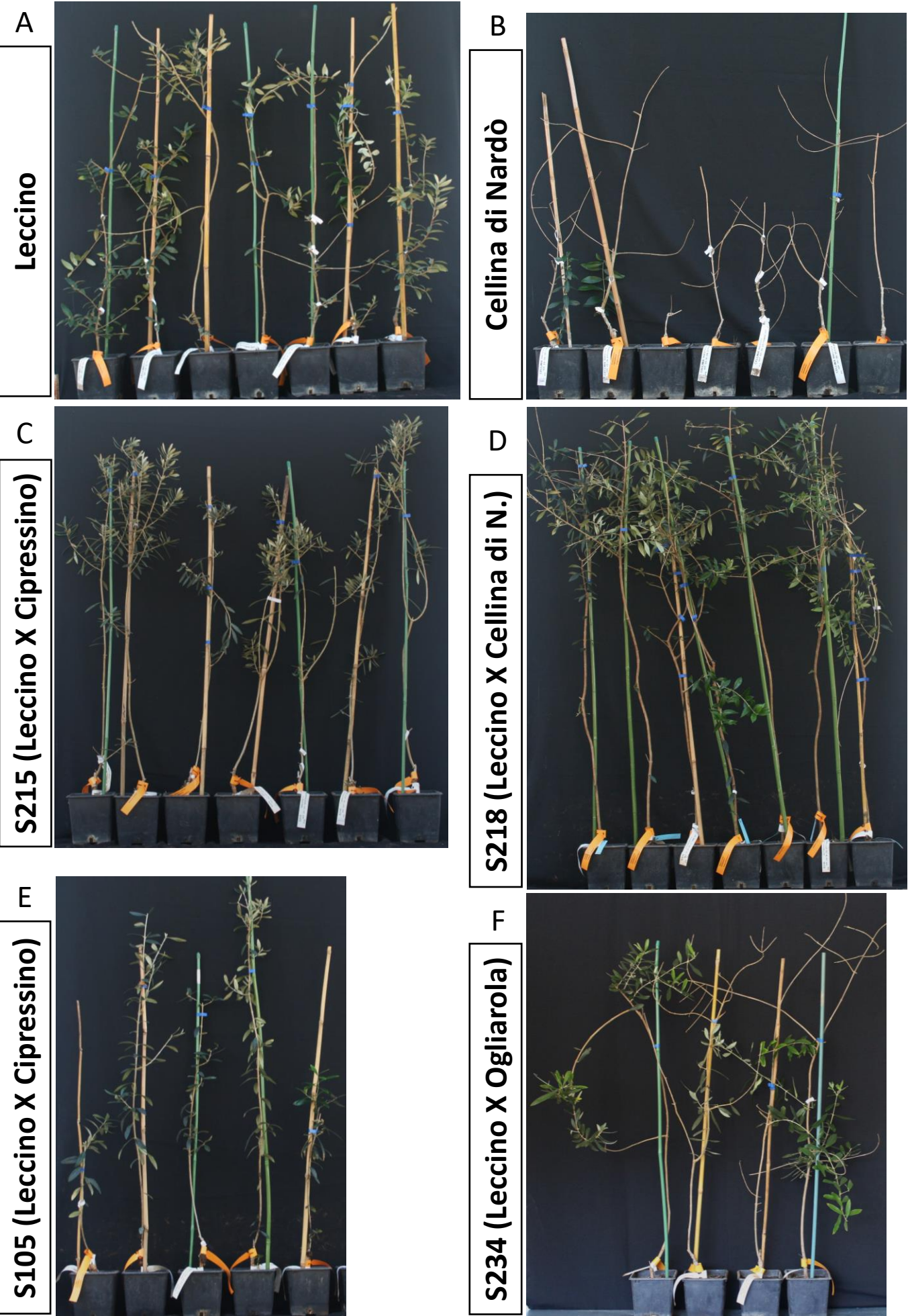

Supplement: Supplementary file 10 [file Presentation9.pdf]
