# Supplementary material for: A survey in natural olive resources exposed to high inoculum pressure indicates the presence of traits of resistance to Xylella fastidiosa in Leccino offspring
Source: Front Plant Sci. 2024 Sep 30;15:1457831. doi: 10.3389/fpls.2024.1457831 (PMC11471571; doi:10.3389/fpls.2024.1457831)
Supplement: Supplementary file 9 [file Presentation8.pptx]

## Slide 1
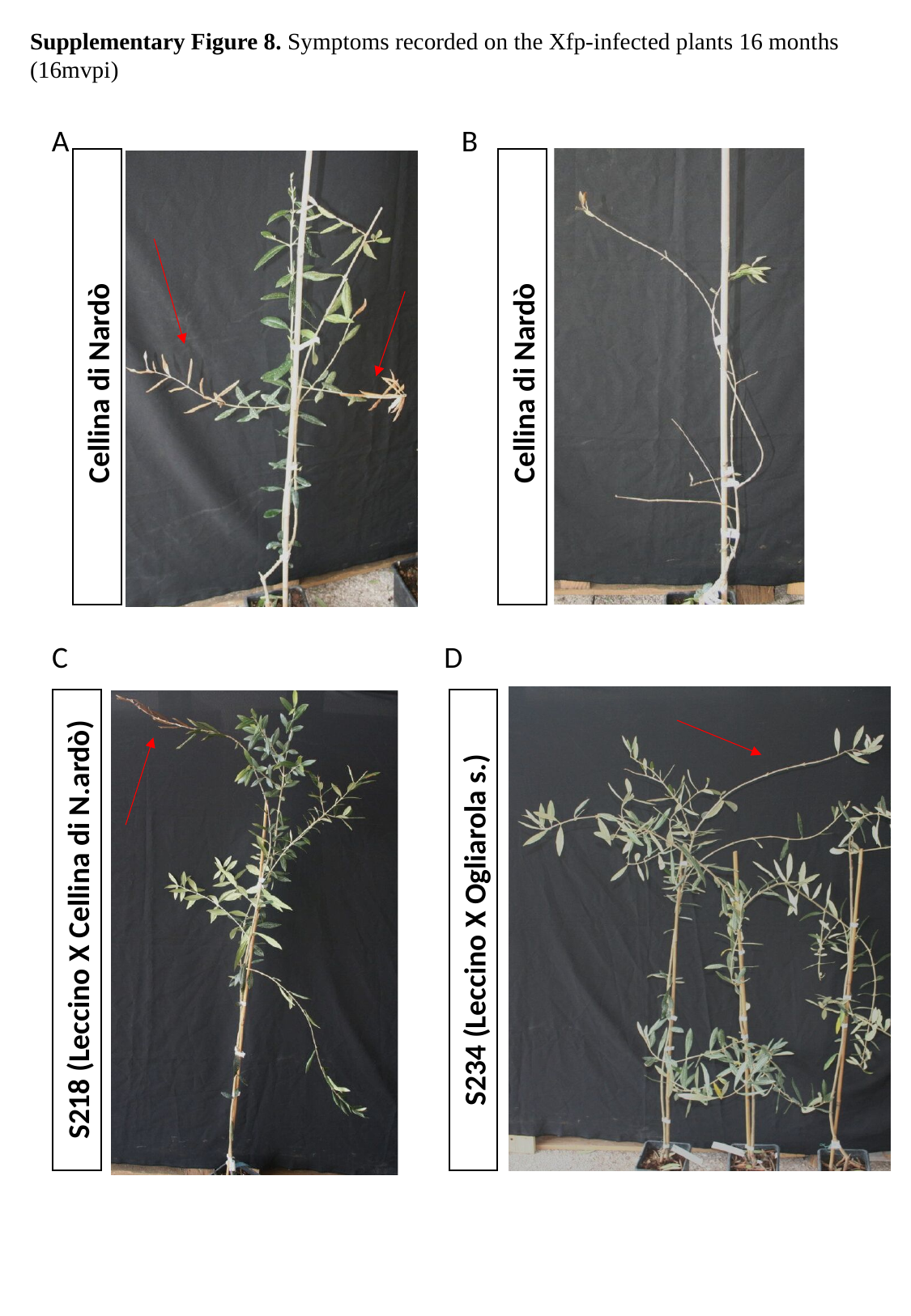

Supplementary Figure 8. Symptoms recorded on the Xfp-infected plants 16 months (16mvpi)
A
B
Cellina di Nardò
Cellina di Nardò
C
D
S234 (Leccino X Ogliarola s.)
S218 (Leccino X Cellina di N.ardò)
