# Supplementary material for: A survey in natural olive resources exposed to high inoculum pressure indicates the presence of traits of resistance to Xylella fastidiosa in Leccino offspring
Source: Front Plant Sci. 2024 Sep 30;15:1457831. doi: 10.3389/fpls.2024.1457831 (PMC11471571; doi:10.3389/fpls.2024.1457831)
Supplement: Supplementary file 11 [file Table1.docx]

| Known cultivars | HR | HS | R | S | T | Total individual trees |
| --- | --- | --- | --- | --- | --- | --- |
| Arbequina |  |  | 1 |  |  | 1 |
| Ascolana tenera |  |  |  | 3 | 2 | 5 |
| Canino |  |  | 1 |  |  | 1 |
| Carolea |  |  |  | 1 |  | 1 |
| Cellina di Nardò |  | 4 |  | 1 |  | 5 |
| Coratina |  |  |  | 1 |  | 1 |
| Gaydourelia |  |  | 1 |  |  | 1 |
| Leccino | 1 |  | 7 |  | 1 | 9 |
| Nocellara messinese |  |  |  | 1 |  | 1 |
| Ogliarola salentina |  | 1 |  | 1 | 1 | 3 |
| Pendolino |  |  | 1 |  |  | 1 |
| Simone |  |  | 1 |  |  | 1 |
| Uovo di piccione |  |  |  | 1 |  | 1 |
| Vasilikada |  |  |  |  | 1 | 1 |
| Total | **1** | **5** | **12** | **9** | **5** | **32** |

**Supplementary Table 1**. Categorization of the selected trees belonging to known cultivars.
