# Supplementary material for: A survey in natural olive resources exposed to high inoculum pressure indicates the presence of traits of resistance to Xylella fastidiosa in Leccino offspring
Source: Front Plant Sci. 2024 Sep 30;15:1457831. doi: 10.3389/fpls.2024.1457831 (PMC11471571; doi:10.3389/fpls.2024.1457831)
Supplement: Supplementary file 12 [file Table2.docx]

**Supplementary Table 2.** Indices of genetic diversity of 171 genotypes for each SSR locus: number of alleles (Na); number of effective alleles (Ne); Shannon’s information index (I); observed heterozygosity (Ho); expected heterozygosity (He); fixation index (F); Polymorphism Information Content (PIC) and null alleles frequency F(Null).

| **Locus** | **Na** | **Ne** | **I** | **Ho** | **He** | **F** | **PIC** | **F(Null)** |
| --- | --- | --- | --- | --- | --- | --- | --- | --- |
| **DCA3** | 12 | 5.26 | 1.91 | 0.83 | 0.81 | -0.03 | 0.79 | -0.01 |
| **DCA5** | 11 | 3.24 | 1.46 | 0.75 | 0.69 | -0.09 | 0.64 | -0.05 |
| **DCA9** | 21 | 6.52 | 2.22 | 0.94 | 0.85 | -0.11 | 0.83 | -0.06 |
| **DCA16** | 20 | 5.23 | 2.06 | 0.84 | 0.81 | -0.04 | 0.79 | -0.02 |
| **DCA18** | 17 | 5.70 | 2.05 | 0.90 | 0.82 | -0.09 | 0.81 | -0.05 |
| **EMO90** | 5 | 2.72 | 1.16 | 0.64 | 0.63 | -0.01 | 0.56 | -0.02 |
| **GAPU71B** | 8 | 2.94 | 1.31 | 0.67 | 0.66 | -0.02 | 0.60 | -0.02 |
| **GAPU101** | 14 | 4.60 | 1.82 | 0.88 | 0.78 | -0.12 | 0.76 | -0.06 |
| **GAPU103A** | 19 | 4.69 | 1.89 | 0.73 | 0.79 | 0.08 | 0.76 | 0.04 |
| **UDO-043** | 19 | 6.82 | 2.12 | 0.78 | 0.85 | 0.08 | 0.84 | 0.04 |
| **Mean** | 146 | 4.77 | 1.80 | 0.80 | 0.80 | -0.03 | 0.74 | -0.02 |
