# Supplementary material for: A survey in natural olive resources exposed to high inoculum pressure indicates the presence of traits of resistance to Xylella fastidiosa in Leccino offspring
Source: Front Plant Sci. 2024 Sep 30;15:1457831. doi: 10.3389/fpls.2024.1457831 (PMC11471571; doi:10.3389/fpls.2024.1457831)
Supplement: Supplementary file 16 [file Table6.docx]

**Supplementary Table 16.**

| **Primer name** | **Oligonucleotide sequence 5'-3'** |
| --- | --- |
| **Primers for Simple Sequence Repeat (SSR) genotyping *** | |
| DCA3 FW^a^ | CCCAAGCGGAGGTGTATATTGTTAC |
| DCA3 REV^a^ | TGCTTTTGTCGTGTTTGAGATGTTG |
| DCA5 FW^a^ | AACAAATCCCATACGAACTGCC |
| DCA5 REV^a^ | CGTGTTGCTGTGAAGAAAATCG |
| DCA9 FW^a^ | AATCAAAGTCTTCCTTCTCATTTCG |
| DCA9 REV^a^ | GATCCTTCCAAAAGTATAACCTCTC |
| DCA16 FW^a^ | TTAGGTGGGATTCTGTAGATGGTTG |
| DCA16 REV^a^ | TTTTAGGTGAGTTCATAGAATTAGC |
| DCA18 Fw^a^ | AAGAAAGAAAAAGGCAGAATTAAGC |
| DCA18 REV^a^ | GTTTTCGTCTCTCTACATAAGTGAC |
| UDO99–043 Fw^b^ | TCGGCTTTACAACCCATTTC |
| UDO99–043 REV^b^ | TGCCAATTATGGGGCTAACT |
| GAPU71B Fw^c^ | GATCAAAGGAAGAAGGGGATAAA |
| GAPU71B REV^c^ | ACAACAAATCCGTACGCTTG |
| GAPU101 Fw^c^ | CATGAAAGGAGGGGGACATA |
| GAPU101 REV^c^ | GGCACTTGTTGTGCAGATTG |
| GAPU103A Fw^c^ | TGAATTTAACTTTAAACCCACACA |
| GAPU103A REV^c^ | GCATCGCTCGATTTTTATCC |
| EMO90 FW^d^ | CATCCGGATTTCTTGCTTTT |
| EMO90 REV^d^ | AGCGAATGTAGCTTTGCATGT |
| *Note: the original name and the relative sequence was reported for each marker. | |
|  | |
| **Primers and probes for diagnostic test for *Xfp* by real time quantitative PCR (qPCR)** | |
| Bacterial rimM gene^e^ (Harper et al., 2010) | |
| XF-F | CACGGCTGGTAACGGAAGA |
| XF-R | GGGTTGCGTGGTGAAATCAAG |
| XF-P | 6-FAM -TCGCATCCCGTGGCTCAGTCC-BHQ-1 |
| Plant cytochrome oxidase (COX) gene^f^ (Weller et al., 2000) | |
| COX-F | CGT CGC ATT CCA GAT TAT CCA |
| COX-R | CAA CTA CGG ATA TAT AAG AGC CAA AAC TG |
| COX-P | Cy5-TGC TTA CGC TGG ATG GAA TGC CCT-BHQ-2 |

1. Sefc, K.M.; Lopes, M.S.; Mendonca, D.; Dos Santos, M.R.; Laimer, M.; da Câmara Machado, A. Identification of microsatellite loci in olive (Olea europaea) and their characterization in Italian and Iberian olive trees. Mol. Ecol. 2000, 9, 1171–1173.
2. Cipriani, G.; Marrazzo, M.T.; Marconi, R.; Cimato, A.; Testolin, R. Microsatellite markers isolated in olive (Olea europaea L.) are suitable for individual fingerprinting and reveal polymorphism within ancient cultivars. Theor. Appl. Genet. 2002, 104, 223–228.
3. Carriero, F.; Fontanazza, G.; Cellini, F.; Giorio, G. Identification of simple sequence repeats (SSRs) in olive (Olea europaea L.). Theor. Appl. Genet. 2002, 104, 301–307.
4. De la Rosa, R.; James, C.M.; Tobutt, K.R. Isolation and characterization of polymorphic microsatellite in olive (Olea europaea L.) and their transferability to other genera in the Oleaceae. Mol. Ecol. 2002, 2, 265–267.
5. Harper, S. J., Ward, L. I., & Clover, G. R. G. (2010). Development of LAMP and real-time PCR methods for the rapid detection of Xylella fastidiosa for quarantine and field applications. *Phytopathology*, *100*(12). https://doi.org/10.1094/PHYTO-06-10-0168
6. Weller, S. A., Elphinstone, J. G., Smith, N. C., Boonham, N., & Stead, D. E. (2000). Detection of Ralstonia solanacearum strains with a quantitative, multiplex, real-time, fluorogenic PCR (TaqMan) assay. *Applied and Environmental Microbiology*, *66*(7). https://doi.org/10.1128/AEM.66.7.2853-2858.2000
